# Two-photon interference at telecom wavelengths for time-bin-encoded single photons from quantum-dot spin qubits

**DOI:** 10.1038/ncomms9955

**Published:** 2015-11-24

**Authors:** Leo Yu, Chandra M. Natarajan, Tomoyuki Horikiri, Carsten Langrock, Jason S. Pelc, Michael G. Tanner, Eisuke Abe, Sebastian Maier, Christian Schneider, Sven Höfling, Martin Kamp, Robert H. Hadfield, Martin M. Fejer, Yoshihisa Yamamoto

**Affiliations:** 1 E. L. Ginzton Laboratory, Stanford University, 348 Via Pueblo Mall, Stanford, California 94305, USA; 2 National Institute of Informatics, Hitotsubashi 2-1-2, Tokyo 101-8403, Japan; 3 School of Engineering, University of Glasgow, Glasgow G12 8QQ, Scotland, UK; 4 Yokohama National University, 79-5 Tokiwadai, Hodogaya, Yokohama 240-8501, Japan; 5 Hewlett-Packard Laboratories, 1501 Page Mill Road, Palo Alto, California 94304, USA; 6 Scottish Universities Physics Alliance (SUPA) and School of Engineering and Physical Sciences, Heriot-Watt University, Edinburgh EH14 4AS, UK; 7 Technische Physik, Physikalisches Institut and Wilhelm Conrad Röntgen-Center for Complex Material Systems, University of Würzburg, Am Hubland, Würzburg 97074, Germany; 8 School of Physics and Astronomy, University of St Andrews, St Andrews KY16 9SS, UK

## Abstract

Practical quantum communication between remote quantum memories rely on single photons at telecom wavelengths. Although spin-photon entanglement has been demonstrated in atomic and solid-state qubit systems, the produced single photons at short wavelengths and with polarization encoding are not suitable for long-distance communication, because they suffer from high propagation loss and depolarization in optical fibres. Establishing entanglement between remote quantum nodes would further require the photons generated from separate nodes to be indistinguishable. Here, we report the observation of correlations between a quantum-dot spin and a telecom single photon across a 2-km fibre channel based on time-bin encoding and background-free frequency downconversion. The downconverted photon at telecom wavelengths exhibits two-photon interference with another photon from an independent source, achieving a mean wavepacket overlap of greater than 0.89 despite their original wavelength mismatch (900 and 911 nm). The quantum-networking operations that we demonstrate will enable practical communication between solid-state spin qubits across long distances.

## Abstract (web summary)

Quantum communication requires quantum correlations between the information processing units and the information carrying units. Here, the authors use time-bin encoding and frequency downconversion to telecom wavelengths to achieve kilometre-scale spin-photon correlations.

## Graphical abstract

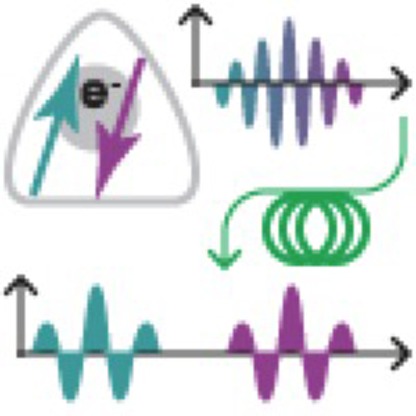

Quantum physics can empower current network infrastructures with fundamentally new functionalities^1^ such as quantum key distribution, distributed quantum computation, quantum clock synchronization and very-long-baseline interferometry, motivating the research for a quantum internet^2^. Essential to the quantum internet is establishing entanglement between remote quantum nodes, which is practically realized by extending spin-photon entanglement^3^,^4^,^5^,^6^,^7^,^8^ using indistinguishable photons^9^ in Bell state measurements^10^,^11^,^12^,^13^. Once entanglement is established, information can be transferred between the nodes using quantum teleportation. However, in a quantum internet that comprises multifarious nodes and spans long distances, such a protocol may fail because of photon attenuation, which-path information leakage, decoherence and distinguishability. First, photon attenuation over optical fibres worsens at the native wavelengths of quantum nodes with demonstrated quantum computing capability^14^. The attenuation exponentially adds up to more than 30 dB at a node spacing of 10 km, as is commonly assumed in quantum repeater research^1^. Second, photons that are entangled with spins may leak which-path information associated with the energy difference between nondegenerate spin states. The eventual registration of photons then causes the spin states to mix incoherently. Third, photon polarization in standard optical fibres changes uncontrollably owing to birefringence, which is unavoidable in practice because of stress and temperature variations and renders the common polarization encoding of photonic qubits^3^,^4^,^5^,^6^,^7^ prone to decoherence^15^,^16^. Converting orthogonally polarized photons with equal efficiencies would also require nontrivial engineering of frequency-conversion devices that are generally polarization dependent. Fourth, photon distinguishability due to the heterogeneity among quantum nodes hinders two-photon interference that is crucial to the remote establishment of entanglement; this problem has motivated research on both intrinsic strain^17^ and electric field^18^ tuning as well as extrinsic quantum frequency upconversion^19^,^20^. However, none of these techniques can be generally applied to heterogeneous quantum nodes while simultaneously achieving long communication distances.

In the following, we address these challenges by employing four operations that are broadly identifiable as quantum networking (Fig. 1): wavelength conversion, quantum erasure, time-bin encoding and mediated two-photon interference. A quantum dot (QD) generates an entangled spin-photon pair^7^,^8^, whose propagating photonic component is then time-bin encoded. Both basis states of the time-bin qubit can be supported by a single quantum frequency downconverter, which allows for wavelength conversion to telecom wavelengths and concurrently for quantum erasure^7^ of the which-path information in photon energy using ultrafast/broadband pulses^7^. These time-bin-encoded photons at telecom wavelengths propagate in optical fibres with minimal loss and decoherence, coalescing with other photons of heterogeneous origins to exhibit two-photon interference as a result of the mediation provided by the downconverters, which render them indistinguishable in terms of wavelength and wavepacket. Upon a successful Bell state measurement, the QD spin states can eventually be swapped to a remote spin or photon. Of these four central quantum-networking operations, we have previously demonstrated the former two^7^,^21^; in this work, we demonstrate time-bin encoding and mediated two-photon interference.

## Results

### Overview

Starting from a QD as the end point of a quantum network, we demonstrate spin-photon correlations that relate a spin qubit to a photonic time-bin qubit, single-photon downconversion that facilitates a photon to transfer quantum information, and two-photon interference between a QD photon and another photon that originates from an independent source, all through telecom wavelengths.

### Spin-time-bin correlations

Our demonstration of long-distance spin-photon correlations was achieved based on charged InAs QDs in a magnetic field, which served as a source of entangled spin-photon pairs. Their propagating photonic components would be time-bin encoded and downconverted to preserve their correlations with the QD spins across long distances. In the Voigt geometry, in which the magnetic field is perpendicular to the growth direction/optical axis, the charged QDs have the level structure shown in Fig. 2a. The level structure can be inferred from their magneto-photoluminescence spectrum, shown in Fig. 2b. The ground states contain an electron, and the excited states are the trion states, in which the added electron-hole pair forms a three-body composite with the original electron. Each trion state is optically connected to the two underlying electron spin states, thereby forming a so-called Λ system, which constitutes the basis for all-optical spin control^22^. Moreover, the QD photon that is spontaneously emitted from the excited state has been shown to be entangled with the QD spin^7^,^8^; the combined spin-photon state is written in equation (1).


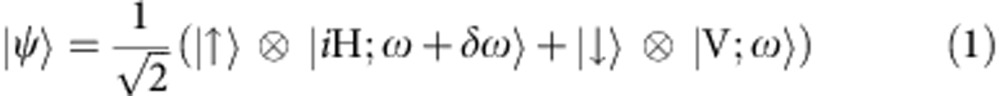



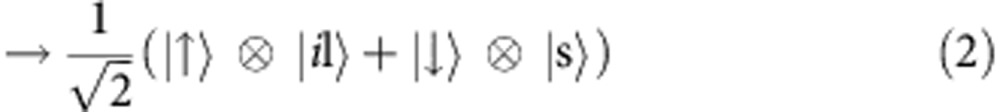


The entangled spin-photon pair represented in equation (1) was generated from a QD in the following sequence: the QD spin was first initialized into the 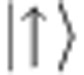 state through optical pumping on the 
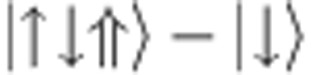
 transition and was then rotated to the 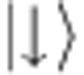 state with a 4-ps-long, 1-nm red-detuned, σ^+^-polarized optical pulse. A third optical pulse, which was 30 ps long and on resonance with the 
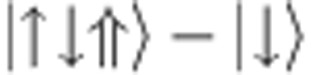
 transition, excited and triggered the QD to spontaneously emit the entangled photon, which could be subsequently downconverted to extend its propagation distance. This sequence concluded with spin readout, which was also performed via optical pumping and whose projection basis could be changed to 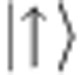 by applying an additional preceding spin-rotation pulse.

Our quantum downconverter, illustrated in Fig. 4a, frequency downconverted photons in the signal wavelength range of *λ*_s_=900–911 nm to a target wavelength *λ*_t_ in the telecom L-band (1,565−1,625 nm). The core operation, a difference-frequency generation (DFG) process in a periodically poled lithium niobate (PPLN) waveguide^21^, was pumped by a strong pulse that was independently tunable in terms of wavelength and wavepacket. The pump pulse allowed translation of *λ*_t_ by more than 30 nm. Unlike a continuous-wave (c.w.) pump, this pulsed pump could be used to shape the target wavepacket either generically or specifically to a squared hyperbolic secant (sech^2^) shape with nominal settings of 30 or 100 ps for our subsequent demonstrations. The pump pulse gained sufficient power for downconversion from the holmium-doped fibre amplifiers that we adopted (Methods). We present further studies of the downconverter later in the text.

The entanglement that existed between the QD spin and the photon polarization was encoded into the spin-time-bin format shown in equation (2) using the two interferometers depicted in Fig. 3a. An unbalanced polarization Michelson interferometer introduced a 1.2-ns difference in the optical path length (OPL) between the QD H- and V-photons. Correspondingly, the pump pulses for downconversion were also passed through an unbalanced fibre interferometer with the same OPL difference, prepared in both time bins to downconvert the QD photons. The downconverted QD photons in the s and l bin are shown in Fig. 3b together with the downconverted laser leakage, which acted as background noise. The actual QD signal was identified by comparing the cases with the spin-rotation pulse switched on and off; the absence of this pulse would cause the spin to be trapped in the 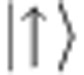 state and prohibit further spontaneous emission. By taking the difference between the two traces shown in Fig. 3b, we estimated the signal-to-noise ratio to be 1–3 under various experimental conditions. The downconverted laser leakage originated from the finite extinction of the spin-initialization pulses and entanglement-generation pulses in their nominal ‘off' states (Methods).

We measured the spin-photon correlations in different bases to confirm their persistence after transmission through a 2-km fibre link. Before downconversion, a strong anti-correlation between the spin 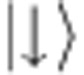 state and the photon H state was observed (Fig. 3d), in agreement with equation (1); the conditional probability 
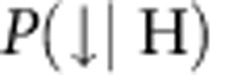
 after correction for memory effects was 0.02±0.00 (Methods). The V polarization was not measured concurrently because of the strong reflection from the resonant, entanglement-generation pulse. After being encoded into the time-bin format and downconverted, the QD photons in orthogonal states (s and l bins) could be measured concurrently because the reflection from the entanglement-generation pulse was largely avoided via time gating: the downconversion pulse was 30 ps long and delayed with respect to the reflection by 400 ps (Fig. 3b). In agreement with equation (1), (anti-)correlation between the spin 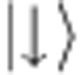 state and the photon (l) s state was observed (Fig. 3e, f). The conditional probabilities 
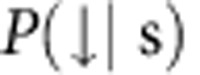
 and 
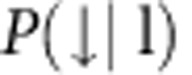
 were 1.25±0.13 and 0.14±0.03, respectively, without correction; the latter deviated from 
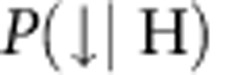
 because the downconverted laser leakage resulted in false photon-detection events that were uncorrelated with the QD spin state. After correction for memory effects^23^ and laser leakage (Methods), the probabilities were 1.16±0.14 and 0.01±0.03, respectively. For completeness, we also measured the correlations between the spin 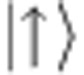 state and the photon H, s, and l states by applying an additional spin-rotation pulse before spin readout. Although these measurements further suffered from unwanted trion excitation by the spin-rotation pulse, which lowered the fidelity of the subsequent spin readout^8^, they were not essential to the entanglement-swapping protocol. The overall correlation results, summarized in Fig. 3c, confirmed the persistence of spin-time-bin correlations after transmission through the 2-km fibre link.

### Background-free downconversion

The communication of the QD with the next node was further facilitated using the downconverter, which could improve photon statistics and indistinguishability and mediate two-photon interference. We quantify these effects in the low-photon-number limit: A quantum state of light, with its photon wavepacket given as *x*(*t*), can be approximated as 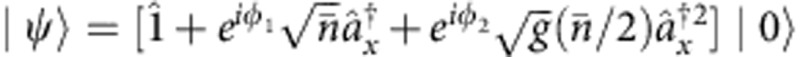, where 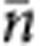 is its mean photon number and *g* measures its two-photon probability relative to that of a Poissonian source. The photon wavepacket creation operator, defined as 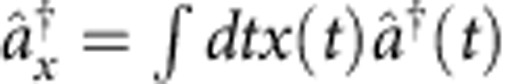 with 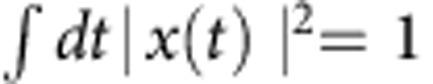, satisfies the commutation relation 
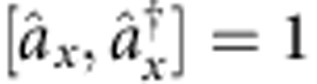
. The mean wavepacket overlap, 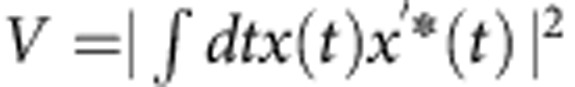, measures the indistinguishability of photons among one another, and vanishes between photons unless they are matched in terms of both wavelength and wavepacket. When the quantum states of light are not pure, the mean wavepacket overlap can readily be shown to become 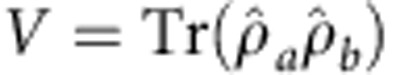, where 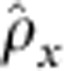 is the density operator of the quantum state *x*. The overlap *V* approaches 1 only when both 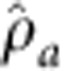 and 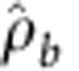 are in pure states, in which case this generalization agrees with the original definition of *V* (see Supplementary Note 1 for further discussion).

Practically, to verify that single photons could be downconverted with high efficiency and without background noise, a QD was excited via *p*-shell excitation at a wavelength of 6 nm below its main emission line at 910.85 nm (Fig. 5a). The QD photons exhibited an exponentially decaying pulse shape with a time constant of 681 ps, which were then downconverted to a wavelength of 1,610 nm and a sech^2^-shaped pulse with a time constant of 120 ps, as shown in Fig. 5b and verified in a cross-correlation measurement (Fig. 5c; Methods). The downconverter generated 34.9k photon counts per second (c.p.s.) with 88% conversion efficiency; the overall system efficiency, 5.3%, was determined primarily by the finite transmission and the pulse-duration mismatch (Methods). While converting a single-photon-level signal, the downconverter did not generate observable background noise; this noise level could be precisely quantified with the noise-count probability per pump pulse at peak downconversion. We lowered this probability from the level of 10^−8^ in our previous study^21^ to less than 10^−11^. This low level of probability represents the demonstration of background-free quantum frequency downconversion (QFDC; Methods).

By applying background-free downconversion, the quantum downconverter could improve the photon statistics and indistinguishability of a single-photon source, which ultimately determine the fidelity to transfer quantum information. The QD photons, both before and after downconversion, were directed to Hanbury Brown–Twiss (HBT) setups for photon-correlation measurements (Fig. 5e). Extracted from the resulting histograms (Fig. 5e), the second-order autocorrelation function at zero time delay, *g*^(2)^(0), was improved from 0.11±0.01 to 0.03±0.02 before and after downconversion, respectively. To test the photon indistinguishability, consecutive photons^24^ were generated from the QD and subjected to two-photon interference before and after downconversion, as shown in Fig. 4b. In the resulting time-correlated photon coincidence measurements shown in Fig. 5f, the diminished centre peaks (at a delay of 0 ns) correspond to the coincidence events that occurred when the leading (single) photon followed the long arm of the interferometer and the trailing (single) photon followed the short arm. In these events, the two photons coalesced at the second beamsplitter and were thus less likely to generate a coincidence. Further analysis based on the relative peak areas showed that the mean wavepacket overlap, *V*, was improved from 0.73±0.04 to 0.89±0.07 before and after downconversion, respectively (Methods). We attribute this improvement in photon statistics and indistinguishability to a time-filtering effect in the mixing of the pump pulse with the QD photons: the short pump pulses downconverted comparatively less of the unwanted background emission than they did of the QD emission, which jittered less in time; the broader bandwidth of the pump pulses also improved *V* by dominating the pure dephasing rate of the QD photons (Methods).

### Mediated two-photon interference

Moving on to a heterogeneous quantum network, we generalize previous treatments^18^,^25^ of two-photon interference and mediate it using quantum downconverters. For two nodes *a*, *b* that generate quantum states of light with general *g* and wavepackets, we derived the normalized photon coincidence number (Methods):


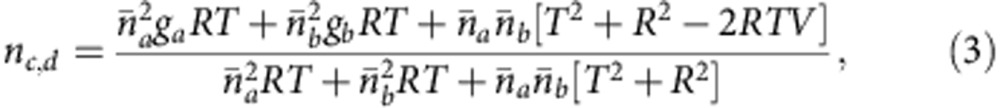


where *R* and *T* are the reflection and transmission intensity coefficients of the beamsplitter. In the simplest case of *R*=*T*=1/2, 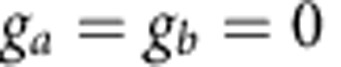 and *V*=1, *n*_*c,d*_ goes to zero. That is, two indistinguishable single photons coalesce at a 50/50 beam splitter without generating a coincidence. More generally, both the numerator and denominator of *n*_*c,d*_ can be interpreted as a probability sum of two-photon and one-photon events weighted by the probability associated with the paths that the photons take. As long as *V* is nonzero, two-photon interference results in a reduction of *n*_*c,d*_. Also commonly quoted in the literature is the two-photon interference visibility, which follows from equation (3): 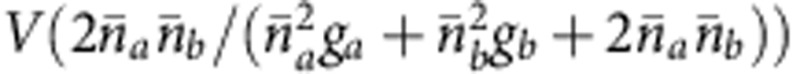 when *R*=*T*. The visibility does not exceed *V* but may further degrade because of finite *g*; this effect, however, could be offset by decreased 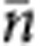.

Quantum downconverters can render photons largely indistinguishable regardless of the original separation between the photon sources in terms of wavelength, wavepacket, and distance. For illustration, an attenuated c.w. diode laser was used as a second photon source, in addition to the 911-nm QD. The laser emitted at 900 nm and had a nominal 1-MHz bandwidth, which was at least two orders of magnitude smaller than the QD bandwidth. Single photons from the QD and diode laser were downconverted via two quantum downconverters that were separated by a 2-km fibre, as shown in Fig. 4c, to nearly the same wavelength, as shown in the high-resolution spectra presented in Fig. 6a. From the spectra (Fig. 6a), bandwidths of 7.10 and 4.46 GHz could be extracted for the downconverted QD and laser photons, respectively, based on a Gaussian fit. As naively estimated from the transform-limited time-bandwidth product, the bandwidth of the pump pulse (2.62 GHz) dominated those of the QD (0.23 GHz) and the diode laser and thus predominantly determined the convolved spectra of the downconverted photons. Practically, the overall bandwidth was broadened because of the technical noise of the Ti:sapphire laser and may have been further broadened by the long-term spectral diffusion of the QD. The mean wavepacket overlap *V*, generalized to account for the mixed states that were ensemble averaged over the spectral broadening, could theoretically have reached 0.92 based on the measured spectra, in contrast with the original zero overlap in the absence of downconversion.

Consequently, two-photon interference could be mediated between heterogeneous quantum nodes of general photon statistics. The second downconverter tuned the downconverted laser photons to several frequency detuning points; at each of these points, the normalized photon coincidence number *n*_*c,d*_ was extracted from the corresponding histogram measured using the HBT setup. The mean number of downconverted laser photons was set to 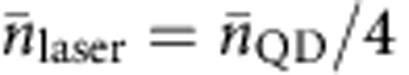 to offset the degradation of visibility because of the finite 
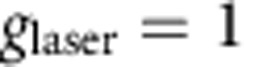
. As shown in Fig. 6b, when the second downconverter eliminated the frequency detuning in the mean wavepacket overlap *V*, a reduction in *n*_*c*,*d*_ was observed as the result of two-photon interference. We arrived at quantitative bounds on the reduction in *n*_*c*,*d*_ by evaluating equation (3) with the experimentally determined 
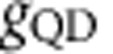
, *V* and spectra (Methods). The measured *n*_*c*,*d*_ overall approached the lower bound closely. At the nominal zero-frequency-detuning point, the overlap *V*, 0.89±0.11, approached the upper bound 0.92; the corresponding two-photon interference visibility, 0.75±0.10, was primarily limited by the finite 
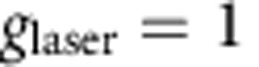
. Based on the high overlap, we conclude that the downconverter effected two-photon interference by further mitigating the degradation because of QD dephasing and long-term spectral diffusion, which has previously limited the overlap *V* to be ∼0.33 or less even between two wavelength-tunable QDs^17^,^18^. We refer the reader to Supplementary Note 2 for further discussion on the possible causes of the nonideal wavepacket overlap.

## Discussion

We have demonstrated correlation between the QD spin and the photon arrival time and mediated two-photon interference at kilometre scales. The next steps towards practical communication between quantum-dot spin qubits readily follow this work. The demonstration of spin-time-bin entanglement would further include measurements in the off-diagonal basis: 
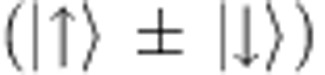
 and 
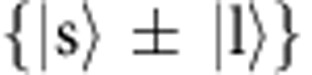
. We have previously demonstrated complete control of a quantum-dot spin over the Bloch sphere^7^,^22^. For time-bin qubits, we designed and realized actively stabilized interferometers (Supplementary Fig. 1; Supplementary Note 3) that maintain the phase relationship between the s- and l-bin photons as they traverse different optical paths during encoding, downconversion and readout. Moreover, our conversion of polarization encoding to time-bin encoding circumvented the nontrivial engineering of frequency-conversion and/or fibre-optic devices that otherwise would be required. (See Supplementary Note 3 for what the nontrivial engineering entails.) The 50% signal loss at the +45° polarizer can be avoided by rotating instead of projecting the polarizations of the s- and l-bin photons using a fast Pockels cell. On the other hand, although our demonstration of mediated two-photon interference involved only one QD, a second QD would not be affected by the multi-photon events and bandwidth mismatch of an attenuated laser, thereby circumventing the condition 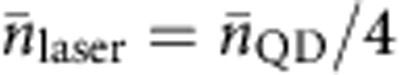 that we set to preserve interference visibility. Mediating the two-photon interference between two quantum nodes is therefore within the demonstrated experimental capability.

Our background-free downconversion results hold broad implications for the applicability of QFDC for quantum communication, ultimately allowing heterogeneous quantum nodes to exchange quantum information across long distances. The range of downconvertible wavelengths, beyond the 11-nm range that we demonstrated, can cover the visible and near-infrared through suitable phase-matching engineering of the frequency converter^26^. Complementarily, the available wavelength range to acquire sufficient pump power has been expanded as a result of our successful adoption of holmium-doped fibre amplifiers, which, in turn, may indicate that other rare-earth-doped fibre amplifiers that are currently under development^27^ may also be suitable for use in quantum optics experiments. The system efficiency can be improved to 27.5% based on our current device by using a longer pump-pulse duration, and even further by optimizing the coupling into PPLN waveguides. Background noise can be essentially eliminated by judiciously choosing the pump wavelength to be sufficiently longer than the target wavelength, and cascaded downconversion^28^ may be adopted to increase wavelength separation if necessary (Supplementary Note 4). Photon coherence, which is implied in our study of photon indistinguishability, renders QFDC useful also in other non-entanglement-swapping-based schemes^29^ for quantum repeaters. Temporal mismatch, which prohibited the efficient downconversion of the laser photons in this work, can be overcome by combining our downconverter with pulse shapers to effect quantum optical waveform conversion^30^.

**Figure 1:**
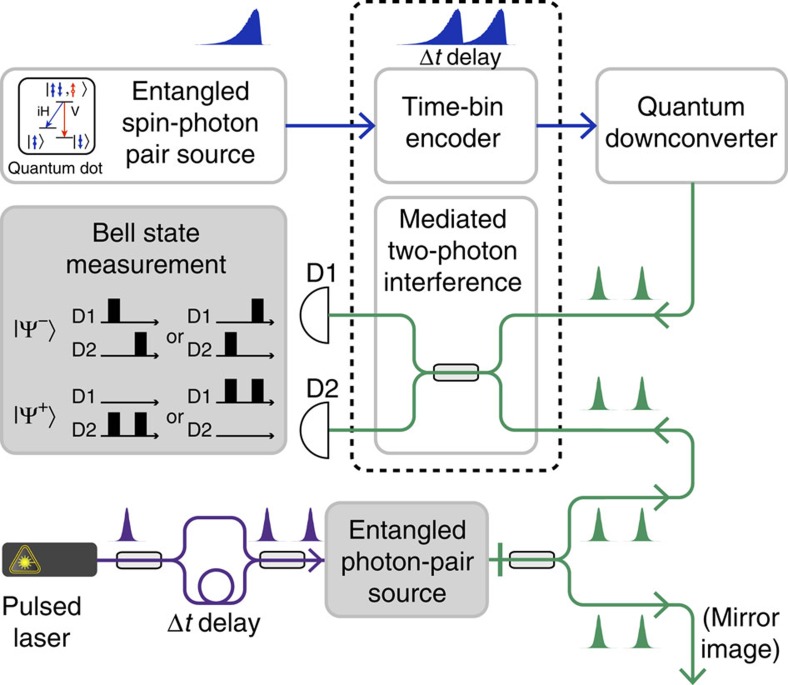
Quantum networking with time-bin encoding and an entangled photon-pair source at midpoint for the entanglement distribution over long distances. After the generation of an entangled spin-photon pair from the quantum dot (QD), the following sequence of operations is performed before the Bell state measurement for entanglement swapping: time-bin encoding, wavelength conversion, quantum erasure and mediated two-photon interference, which can be further assisted with an entangled photon-pair source. In a typical midpoint Bell state measurement scheme, there is another QD at the other end of the quantum channel, and two-photon interference occurs at the midpoint. In the midpoint-source scheme illustrated here, an additional entangled photon-pair source facilitates the speed-up for entanglement distribution, and two-photon interference occurs adjacent to each QD. The adjacency eliminates the communication delay and the dead time for QD operations (see the text). In this figure, the dashed line encircles the main results of this Article, whereas the remaining two plain rectangles and the shaded rectangles indicate our previously achieved and proposed parts, respectively. The experimental implementations corresponding to the four plain rectangles are further shown subsequently in Figs 2e and 3a and Fig. 4a,c, respectively. The entangled photon-pair source is simplified with a semiconductor diode laser in our subsequent demonstration of mediated two-photon interference. The successful coincidence patterns of Bell state measurements in the time-bin basis are shown for concreteness.

The quantum-networking operations may further include entanglement-assisted two-photon interference^31^. In the midpoint-source scheme^31^, an entangled photon-pair source facilitates the speed-up for entanglement distribution, and two-photon interference occurs adjacent to each QD (Fig. 1). The source transmits many photon pairs within one round-trip time window, thereby eliminating the communication delay and the dead time for QD operations. In this scheme, successful Bell state measurements would rely further on two-photon interference being mediated between photons of heterogeneous and even Poissonian origins, an effect that we have demonstrated. Time-bin-entangled photon pairs can be generated from a QD via two-photon resonant excitation^32^ in addition to converting polarization encoding to time-bin encoding, or by pumping a QD^33^ or a PPLN waveguide^34^,^35^ with coherently superposed consecutive laser pulses between which the time delay is longer than the coherence time of the generated photons. We refer the reader to Supplementary Fig. 2 and Supplementary Note 5 for a concrete example.

In summary, we have demonstrated correlation between the QD spin and the photon arrival time, and mediated two-photon interference at kilometre scales. These quantum-networking technologies, together with wavelength conversion^21^ and quantum erasure^7^, will enable practical quantum communication between solid-state spin qubits across long distances.

## Methods

### Quantum-dot spectroscopy and spin control

The QD sample and the low-temperature, magneto-, confocal microscope used in this study were similar to those used previously to investigate spin-photon entanglement^7^; the microscope was based on a 0.68 numerical aperture aspheric lens inside a superconducting magnetic cryostat (Oxford Spectromag). The QD emission was collected into a single-mode fibre that was routed to a spectrometer for spectroscopy, single-photon counting modules (SPCMs) for photon counting or PPLN waveguides for downconversion.

**Figure 2:**
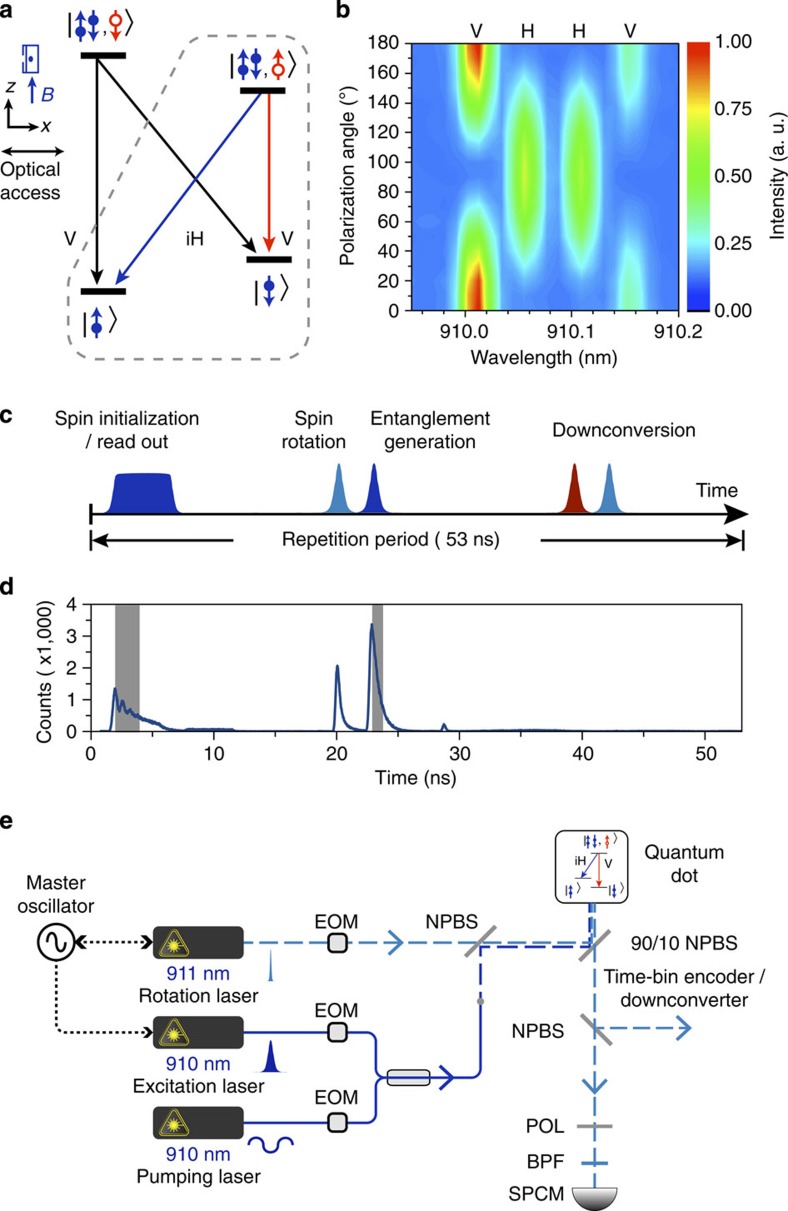
QD as a source of entangled spin-photon pairs. (**a**) Level diagram of a charged InAs QD in Voigt geometry. The dashed line encircles the Λ system that is used to generate entangled spin-photon pairs. In the Voigt geometry, the magnetic field is perpendicular to the growth direction/optical axis. (**b**) Magneto-photoluminescence spectrum of the charged InAs QD under a 6-T magnetic field. (**c**) The optical pulse sequence applied to generate entangled spin-photon pairs from the QD (see the text). (**d**) Photon-counting histogram resulting from the above pulse sequence. The three main features from left to right correspond to single-photon emission during spin readout, residual scattering from the spin-rotation pulse, and QD spontaneous emission, respectively; the last is triggered by the entanglement-generation pulse and features an exponential decay with a time constant of 600 ps. The wavy trace during spin readout is a consequence of the several optical pumping cycles used to fully initialize the QD spin. The shaded areas highlight the time windows for subsequent correlation measurements. (**e**) Experimental setup for generating entangled spin-photon pairs from the QD. The single photons, emitted by the QD and collected from the cryostat, were split by a non-polarizing beamsplitter (NPBS) and directed along two paths. The path for spin readout led to a filtering setup that consisted of an H-polarizer (POL) and a band-pass filter (BPF) with a 1-nm bandwidth. The other path led to the time-bin encoder and, subsequently, to the quantum downconverter. EOM, electro-optic modulators; SPCM, single-photon counting module.

The optical spin-control pulses were generated using three lasers as shown in Fig. 2e: A narrowband c.w. laser (New Focus Velocity), on resonance with the 
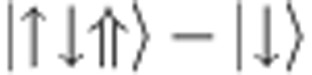
 transition (910.416 nm at 6 T), was used for spin initialization and readout through optical pumping. One Ti:sapphire mode-locked laser (Coherent Mira), centred at 911.42 nm, generated 4-ps pulses to rotate the QD spin. Another Ti:sapphire mode-locked laser (Spectra-Physics Tsunami), which was also used for downconversion, generated 30-ps pulses to excite the QD and trigger entanglement generation. The master clock for the experiment was derived from the Mira laser, to which the Tsunami laser was synchronized through a Spectra-Physics Lok-to-Clock system. The clock was further frequency multiplied to 10 GHz to serve as the reference frequency of a pulse-pattern generator (Anritsu PPG) using a phase-locked frequency synthesizer (Valon Technologies) and an electronic quadrupler (Marki Microwave). The pulse-pattern generator drove fibre-based electro-optic modulators (EOSpace) to shape and pick optical pulses from each laser; in particular, the c.w. laser for spin initialization was externally modulated to produce 4-ns-long pulses.

Reflected laser light was separated from the QD single photons through a combination of spatial, polarization, time and wavelength filtering. The last filtering was implemented using ultra-narrow band-pass filters (Alluxa), which rejected the reflection by at least 4 orders of magnitude at a 1-nm separation and by more than 6 orders of magnitude at large detuning. Resonant laser reflection, which was vertically polarized, was rejected by nearly 60 dB using a crossed polarizer^36^.

The settings of the fibre electro-optic modulator and mode-locked laser were fine-tuned to optimize the extinction of the spin-initialization pulses and entanglement-generation pulses in their nominal ‘off' states. The former pulses had an extinction ratio of ∼33 dB. The latter pulses followed the temporal decay of a sech^2^-shaped pulse only within three times of the pulse duration from the pulse peak, and remained at an extinction level of ∼35 dB even 400 ps after the pulse peak. In the study of spin-photon correlations, the resulting downconverted laser leakage limited the signal-to-noise ratio, which varied from 3 for the spin-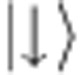 measurement to 1 for the spin-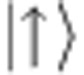 measurement.

### Downconversion

In this subsection, we present the generation and characteristics of the pump pulse. These characteristics allow evaluation of the efficiency of the waveguide DFG process. We close this subsection by summarizing our strategies to eliminate the background noise produced in the downconversion.

The strong pump pulse required in the waveguide DFG process, which was in the wavelength range of 2.04−2.1 μm, was generated using a master oscillator power amplifier configuration. We generated the seed pulses using a DFG process in a bulk MgO-doped PPLN crystal by combining the c.w. light from a 2-W tunable amplified telecom laser with the optical pulses from a Ti:sapphire mode-locked laser (Spectra-Physics Tsunami), whose wavelength was set according to the 
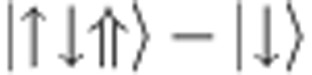
 transition for entanglement generation or at 895 nm in the mediated two-photon interference experiment. After wave mixing, the residual 910/895-nm and telecom light was filtered out using a combination of dichroic and absorptive filters. The resulting seed pulses were coupled into an optical fibre to be further amplified by holmium-doped fibre amplifiers. The amplifier provided a gain of more than 10 dB and a maximum average power of 70 mW in the range 2.04−2.1 μm, thus ensuring that sufficient pump power could be generated to reach peak downconversion under various pulsing conditions. Crucially for single-photon experiments, the amplifier had a large spectral separation between its optical-pumping and gain wavelengths (2.1 μm), thereby allowing for the complete removal of the residual optical-pumping light from the resulting 2.1-μm light using a long-pass filter on a Ge substrate.

The master oscillator power amplifier configuration also provided the downconverter with tunability in terms of pulse duration and wavelength. The pump-pulse duration could be controlled by the Gires–Tournois interferometer of the mode-locked Ti:sapphire laser and was selectable among three nominal settings: 3, 30 and 100 ps. We chose the nominal 30-ps setting for entanglement generation, or the 100-ps setting for mediated two-photon interference when the largest photon yield was desired. The pump wavelength was determined in the bulk DFG process and was therefore tunable through tuning of the telecom laser.

The (2.1-μm) pump pulse characteristics were verified in a cross-correlation measurement, where the 910-nm light was divided to generate 2.1-μm light via DFG inside the bulk PPLN crystal, as well as to inject directly into the waveguide. The 910-nm and 2.1-μm pulses then mixed in the waveguide; the DFG output was monitored while the arrival time of one of the inputs was scanned relative to the other. The cross-correlation results are shown in Fig. 5c for a pulse duration setting of 100 ps. With this setting, the original sech^2^-pulse-shape was largely preserved; a deconvolution indicated a pulse duration of 120 ps.

We used fibre-pigtailed reverse proton-exchange PPLN waveguides to achieve high efficiency for single-photon downconversion experiments^21^ (Supplementary Note 6). In the waveguide DFG process, the nonlinear conversion efficiency *η* depends on the pump power *P*_p_^37^ as follows:


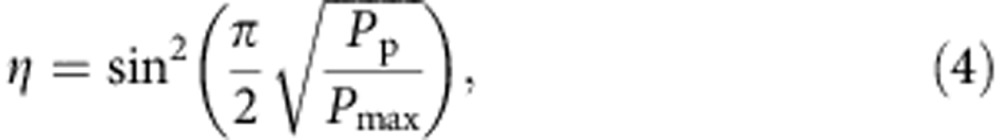


where *P*_max_ is the pump power required for complete conversion. For corresponding illustration, we observed a DFG-signal peak when increasing the pump power as shown in Fig. 5d, thereby determining the sufficiency of pump power for downconversion. Practically, although the entire curve as shown in Fig. 5d (and also as shown in Supplementary Fig. 3) was not taken in every experimental run, a power sweep near the peak was always performed to ensure the most efficient downconversion, which occurred at a peak pump power (in the input fibre) of ∼1 W. We also mention that the data in Fig. 5d do not represent the best coupling of the pump power, therefore *P*_max_ corresponds to a peak pump power higher than 1 W.

We calculated the limit on conversion efficiency because of the duration mismatch between the QD pulse and the pump pulse. For this calculation, the pump pulse shape was substituted into equation (4) to arrive at a time-resolved conversion efficiency, with the assumption that the (instantaneous) efficiency approached unity at the peak of the pump pulse. An overlap integral could then be calculated based on the measured QD pulse shape (in Fig. 5b), and the inferred 120-ps time constant in the cross-correlation measurement of the sech^2^-shaped pump pulse (in Fig. 5c). From this calculation we found the conversion efficiency, limited by pulse duration mismatch, to be 22%, which was notably higher than the ratio between the corresponding time constants, 18%. The difference mainly resulted from the saturation behaviour of equation (4), leading to a conversion window wider than 120 ps.

The overall system downconversion efficiency, which was determined to be 5.3% from the measured 911-nm- and 1,610-nm-photon count rates, was accounted for by other experimentally determined values: the finite time overlap between the QD pulse and the pump pulse (22%) and the transmission efficiency of the waveguide and filters (27.5%), which could be further subdivided into the input coupling and transmission (71%) at 910 nm, the output coupling and transmission (71%) at 1,610 nm, and the filtering setup transmission (55%). We thus estimated that the internal conversion efficiency approached 88%.

To eliminate the background noise produced in the downconversion, we adopted the following strategies: choosing the pump wavelength to be sufficiently longer than the target wavelength^21^, restricting the detection bandwidth, and promptly removing the strong pump light from the downstream optics of the waveguide. The implemented filtering setup, which rejected the pump photons by 18 orders of magnitude in total, included a fibre Bragg grating, a long-pass filter on a Si substrate and a fibre-based 2-μm filter. (See Supplementary Fig. 4 and Supplementary Note 4 for the identification of the origins of the noise.)

### Single-photon counting and data analysis

Two models of SPCMs were used to detect 910-nm single photons. The Micro Photon Devices model (PDM series) had a timing jitter of ∼48 ps and a quantum efficiency of ∼2% at 910 nm and was used to measure the 910-nm photon pulse shape shown in Fig. 5b. Two additional PerkinElmer (SPCM-AQRH-14) SPCMs were used for all other 910-nm photon-counting tasks during the experiment. They both had a quantum efficiency of ∼30% at 910 nm, but they had different timing jitters of ∼430 and ∼780 ps.

The telecom photons were detected using superconducting nanowire single-photon detectors (SNSPDs)^38^. The two SNSPDs, which were maintained at 2 K, had system detection efficiencies of 6 and 20%, un-gated dark count rates of 100 and 200 Hz and full-width-half-maximum timing jitters of 130 and 190 ps. Time-correlated single-photon counting was performed using a time interval analyser (Hydraharp, PicoQuant GmbH) in the time-tagged time-resolved mode. Because the events registered by both the SPCMs and the SNSPDs were accurately time gated during post-processing, the dark counts of the detectors were essentially negligible in this work. The finite number of coincidences, however, resulted in an uncertainty in determining the conditional probabilities as well as *g* and *V* due to Poissonian statistics.

**Figure 3:**
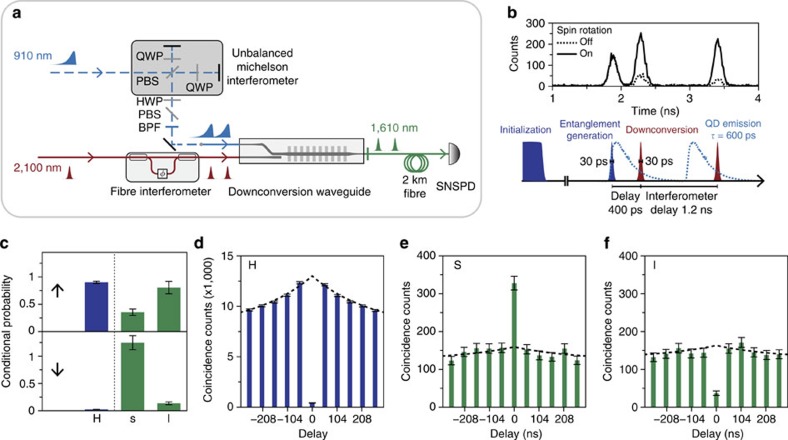
Spin-photon correlation measurements. (**a**) Experimental setup for converting polarization encoding to time-bin encoding. The single photons, emitted by the QD but not directed to spin readout (in Fig. 2e), were directed to a Michelson interferometer and, subsequently, to the downconverter. Shaded area: expanded view of the unbalanced Michelson interferometer. The H- and V-polarized photons, after impinging on the polarizing beamsplitter (PBS), propagated along the long and short arms of the interferometer, respectively, and were then retroreflected at the end mirrors, double passing the quarter-wave plates (QWPs) for the purpose of swapping their polarizations to the orthogonal one (that is, H↔V). After recombining at the PBS, the leading (s-bin) and trailing (l-bin) single photons passed a +45° polarizer (implemented using a half-wave plate (HWP) and a PBS) and departed with only their time bins entangled with the QD spin. The single photons were then coupled into a fibre that connected to the downconverter; before they entered this downconverter, their polarizations were rotated to the extraordinary polarization of the downconverter. (**b**) Top: Occurring in two time bins, the downconverted single photons at telecom wavelength were detected by a superconducting nanowire single-photon detector (SNSPD) at the end of a 2-km fibre link. The difference between the two traces corresponds to the actual single photons from the QD (see the text). Bottom: Arrival time of the single photons and laser scattering in the downconversion waveguide; the downconversion pulse was delayed with respect to the laser scattering to reduce noise. (**c**) Probabilities (uncorrected) of detecting a particular spin basis in 
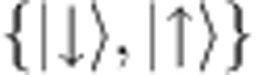
 conditioned upon the detection of a particular photon basis in 
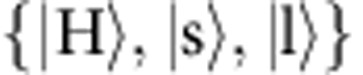
; orthogonal time-bin bases were measured in the same experimental run. (**d**–**f**) Coincidence measurements (**d**) between the spin-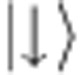 state and the photon-H state, (**e**) between 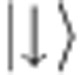 and s and (**f**) between 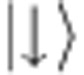 and l. The dashed lines are fits that incorporate the memory effects among single-photon emission from the QD and the laser leakage (Methods). Error bars: ±1 s.d., due to Poissonian statistics.

The spin-photon correlation data were obtained from the histograms (for example, see Fig. 3d–f) of the coincidence counts between the downconverted single photons and the single photons that were used for spin readout. The coincidence counts were obtained through post-processing of the time-tagged time-resolved data stream. In principle, by comparing the coincidence counts registered within the same experimental cycle with those registered in subsequent, uncorrelated cycles, the conditional probability of detecting a spin state given the detection of a photon state could be calculated^7^. For the particular QD that we studied, memory effects resulted in additional positive correlations (blinking^23^) among the QD photon emissions that needed to be corrected for. We fit the binned coincidence counts *C*[*n*] in the delay range of (−528, 1,531.2) ns using a two-sided exponential function plus a background:


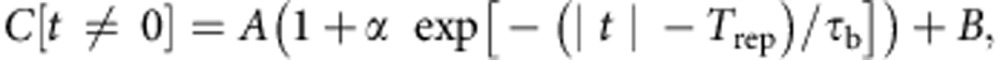


where *T*_rep_ is the period of one experimental cycle (≈52.8 ns); *α* and 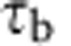 are fitting parameters that characterize the amplitude and time scales, respectively, of the memory effect; *A* accounts for the (uncorrelated) coincidences of actual QD photons; and *B* accounts for the false coincidences caused by the downconverted laser leakage. The extrapolation of the fitting to *C*[*t*→0] then provided a spin-independent factor that could be used to properly normalize the conditional probabilities, which otherwise would have exceeded 1 in the case of 
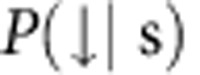
. The ratio of the fitted value of *A* to that of *B* was also found to be consistent with the monitored signal-to-noise ratio preceding each extended run of photon counting. We also verified that the memory effect was not caused by incomplete spin initialization: extending the duration of the spin-initialization pulse from 4 to 13 ns and beyond did not mitigate this effect.

### Two-photon interference

In this subsection, we first summarize the experimental conditions that were common to both of the two-photon interference experiments: the one between consecutive photons and the other between heterogeneous nodes. For the former, we then explain how the reported mean wavepacket overlap *V* was extracted from the histograms in Fig. 5f. For the latter, besides the experimental settings, we explain how the normalized photon coincidence number in equation (6) was derived, and how its reduction was limited by a wavepacket mismatch.

All the two-photon interference experiments were performed using polarization-maintaining fibre beamsplitters to ensure good polarization and mode matching among interfering photons. To generate indistinguishable single photons from the QD, the excitation power was chosen to excite the QD only at 80–90% of its saturation count rate under *p*-shell excitation such that the QD photon coherence could be largely preserved.

For two-photon interference between consecutive photons, the relative peak areas of the five peaks within each central cluster in Fig. 5f were determined by the probability aggregated over the various possible paths that the two photons could take. Each possible path was defined by whether the leading or trailing photon travelled along the short or long arm of the interferometer and then triggered the START or STOP detector. The total number of such possibilities, along with the light properties *g* and *V*, then determined the relative peak areas, as shown in the following equations:


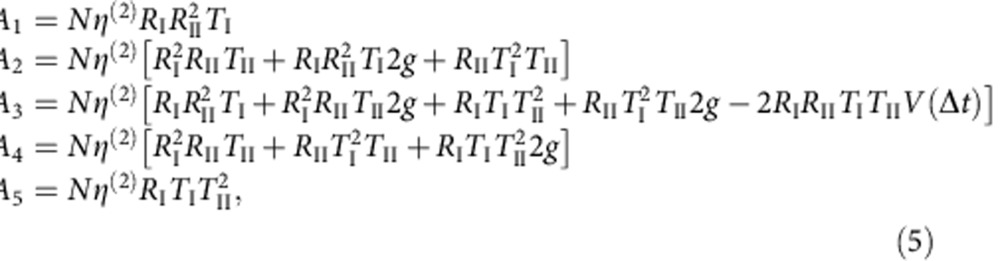


where *N* is the number of repetitions, *η*^(2)^ is the combined efficiency of two-photon generation and detection, and *R*_I_, *R*_II_, *T*_I_ and *T*_II_ are the intensity coefficients of reflection and transmission of the first and second beamsplitters. These equations generalize the previous approach^24^, accounting for the possibility that the two beamsplitters in the Mach–Zehnder interferometer may have different intensity coefficients. The parameter *V*(Δ*t*) in the expression for *A*_3_ represents the mean overlap between the wave packets of the two consecutive photons at a time difference Δ*t*. Using these equations, the overlap *V* between consecutive photons was extracted using the experimentally determined values of *g*, *T*_I_ and *T*_II_.

To apply equation (5) to the data presented in the top panel of Fig. 5f, it was necessary to further extract the areas of individual peaks, which overlapped with one another because of the QD emission tail and the limited time response of the SPCMs used in this experiment. We fit the histogram as a series of peak functions separated by a time delay of 2.6 ns, as illustrated in Supplementary Fig. 5; each peak function, resulting from the START–STOP measurements, was a convolution of a two-sided decaying exponential^39^ (the QD emission) with a Gaussian distribution (of the SPCM time response).

The areas of the five central peak functions could then be used to extract *V* based on equation (5), together with our measured *g*^(2)^(0) in Fig. 5e. In the test of the indistinguishability of consecutive photons before downconversion (corresponding to the top panel of Fig. 5f), the fibre interferometer at 910 nm exhibited *T*_I_=0.77 and *T*_II_=0.43. With the measured *g* being 0.11±0.01, we could estimate *V* at zero time difference to be 0.73±0.04, which was affected by pure dephasing on top of the lifetime-limited bandwidth of the QD photons, 0.23 GHz (see Supplementary Note 2 for further discussion on the possible causes of the nonideal wavepacket overlap). For the test of the indistinguishability of consecutive photons after downconversion (corresponding to the bottom panel of Fig. 5f), we performed a similar estimation and arrived at *V*=0.89±0.07, using the experimentally determined values of *T*_I_=0.50, *T*_II_=0.46 and *g*=0.03±0.02. In addition, we note that the asymmetry among the five peaks in each cluster in Fig. 5f was due to the unbalanced splitting ratio of the fibre beamsplitters.

**Figure 4:**
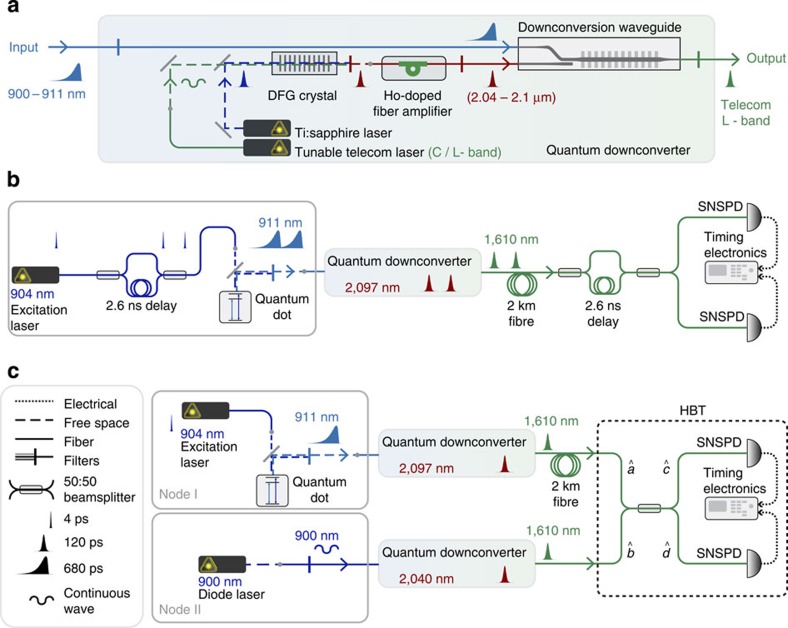
Quantum frequency downconverters and their use in the two-photon interference experiments. (**a**) Schematic of the quantum downconverter highlighting the essential optical elements. Input 900–911 nm photons were downconverted to a target wavelength in the telecom L-band through a difference-frequency generation (DFG) process in the downconversion waveguide. The downconversion process was pumped by a strong pulse that could also translate the target wavelength and shape the target wavepacket. (**b**) The experimental arrangement used to demonstrate two-photon interference between consecutive downconverted QD photons. Twin excitation pulses were prepared using an unbalanced fibre Mach–Zehnder interferometer with an optical-path-length (OPL) difference of 2.6 ns; the twin pump pulses were similarly prepared. (**c**) Experimental arrangement used to demonstrate two-photon interference between downconverted single photons from the QD and from the diode laser. The second downconverter tuned the downconverted laser photons to several frequency detuning points. Dashed rectangle: schematic of an Hanbury Brown–Twiss (HBT) setup. The beamsplitter ports are designated with the operators used in the Methods to derive the normalized photon coincidence number.

**Figure 5:**
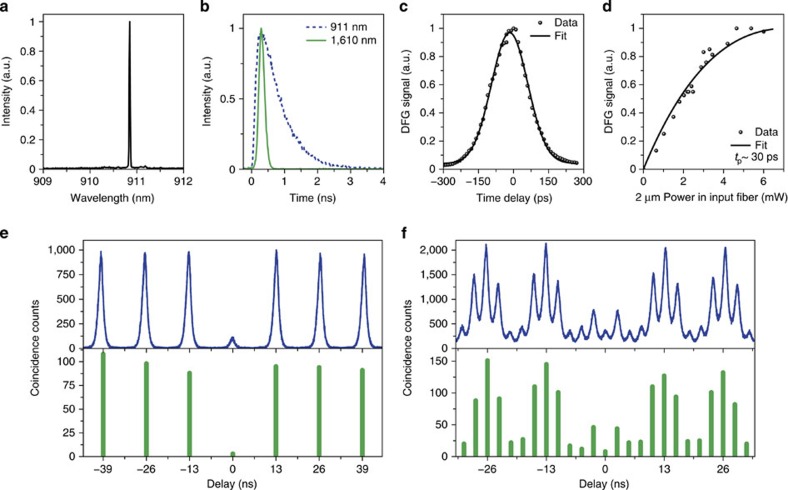
Downconversion of QD single photons. (**a**) Spectrum of QD emission under *p*-shell excitation. (**b**) Pulse shapes of QD single photons before (dotted curve) and after (solid curve). (**c**) Cross-correlation measurements with the pulse duration of the Ti:sapphire mode-locked laser set to 100 ps. (**d**) Pump power dependence of downconversion for illustration. The dots and lines represent, respectively, experimental data and theoretical fits based on equation (4) for the pump pulses of a 30-ps duration. (**e**) Second-order autocorrelation function measurements performed on QD single photons before (top panel) and after (bottom panel) downconversion. (**f**) Two-photon interference of consecutive photons from the QD before (top panel) and after (bottom panel) downconversion, manifesting as the diminished centre peaks at zero time delay (see the text). The data were collected using the setup in Fig. 4b. Each peak in the bottom panels of **e**,**f** corresponds to the aggregated coincidence counts in a time window of 1.3 ns but does not represent their detailed distribution in time.

For two-photon interference between heterogeneous nodes, we used the HBT setup to quantify their properties such as 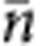, *g* and *V*. The schematic of an HBT setup is shown in Fig. 4c, in which the input ports are labelled as 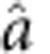, 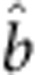 and the output ports as 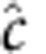, 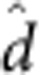. The HBT setup measures the normalized photon coincidence number


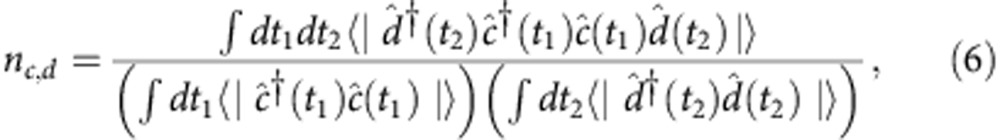


where 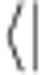 and 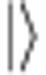 represent the bra and ket of a generic state, respectively. We assume two quantum states of light entering the two ports of the beamsplitter separately, that is,


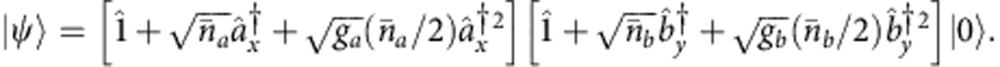


By applying the beamsplitter relationships 
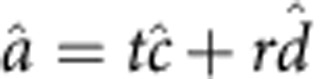
 and 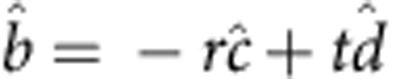, the normalized photon coincidence number as shown in equation (6) is then calculated to obtain equation (3). Equation (3) not only incorporates all the previous scenarios^18^,^25^ in which the interference between two QDs, a QD and a laser, and two lasers have been studied but also treats the cases of finite *g* and super-Poissonian sources, provided that the average photon number is much less than one.

During the QD-laser interference experiment, the sample was temperature stabilized at 2 K to stabilize the QD emission wavelength. To avoid any wavelength mismatch, the wavelengths of all lasers were verified using a wavemeter (Burleigh WA-1100) to within a resolution of 1 pm, and are tabulated in Supplementary Table 1. The spectra of the downconverted photons were further measured using a fibre Fabry–Pérot interferometer with a bandwidth of ∼1 GHz. The second telecom laser was then detuned by ±3 and ±10 GHz, and the normalized photon coincidence number *n*_*c*,*d*_ at each of the corresponding frequencies was extracted from the corresponding histograms, examples of which are provided in Supplementary Fig. 6.

**Figure 6:**
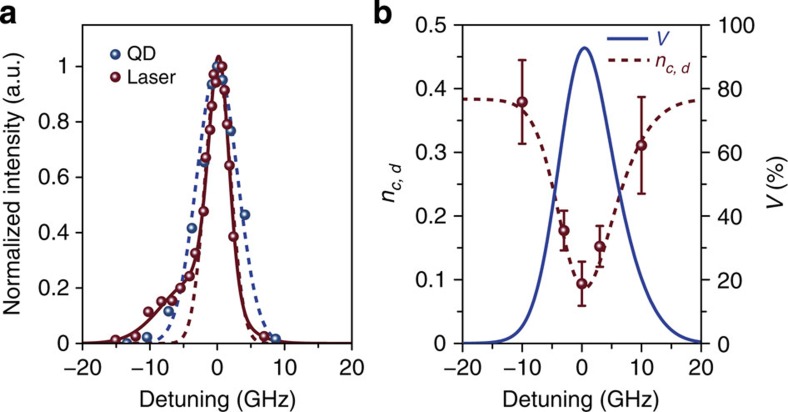
Mediated two-photon interference through downconversion. (**a**) High-resolution spectra of the QD emission (blue dots, raw data; blue dashed line, Gaussian fit) and laser emission (purple–red dots, raw data) after downconversion. The purple–red solid line represents an overall fit to the laser spectrum, whereas the purple–red dashed line is a Gaussian fit to the spectrum with the side lobe arbitrarily removed. (**b**) Normalized photon coincidence number *n*_*c*,*d*_ (purple–red dots, raw data; purple–red dashed line, lower bound) and mean wavepacket overlap *V* (blue solid line, upper bound) in the two-photon interference experiment between downconverted single photons from the QD and from the diode laser. The bounds were obtained by evaluating equation (3) using the experimentally determined 
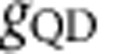
 and the spectra shown in **b** (blue dashed line and purple–red solid line). The data were collected using the arrangement in Fig. 4c. Error bars: ±1 s.d., due to Poissonian statistics (*n*_events_≈50–150).

We analysed the reduction in *n*_*c*,*d*_ in Fig. 6b by evaluating equation (3) using the experimentally determined 
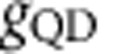
, *V* and spectra to arrive at quantitative bounds. Given only the amplitude but not the phase of the wavepackets from the measured spectra in Fig. 6a, we obtained the following upper bound on the overlap *V* versus the frequency detuning:


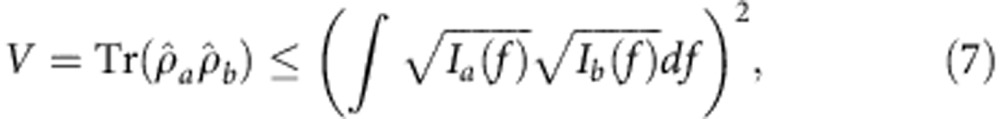


where *I*_*a,b*_(*f*) is the normalized intensity spectrum of 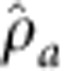, 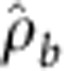 such that 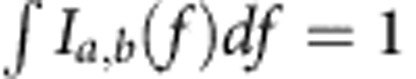. This upper bound is physically motivated by the wavepacket overlap between two (pure) states of constant phase that have individual wavepacket amplitudes of 
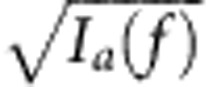
 and 
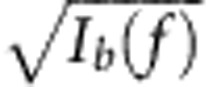
. When the upper bound is approached, dephasing is negligible and the overlap *V* is limited only by the amplitude mismatch between the spectra. This upper bound on the overlap *V* also sets a corresponding lower bound on *n*_*c,d*_ according to equation (3). These bounds, which are also plotted in Fig. 6b, were adjusted only by an overall frequency shift (0.53 GHz) to offset the finite frequency resolution (1 GHz) of the measurement. The measured *n*_*c,d*_ overall approached the lower bound closely.

## Additional information

**How to cite this article:** Yu, L. *et al.* Two-photon interference at telecom wavelengths for time-bin-encoded single photons from quantum-dot spin qubits. *Nat. Commun.* 6:8955 doi: 10.1038/ncomms9955 (2015).

## Supplementary Material

Supplementary Information Supplementary Figures 1-6, Supplementary Table 1, Supplementary Notes 1-6 and Supplementary References
