# Supplementary material for: Two-photon interference at telecom wavelengths for time-bin-encoded single photons from quantum-dot spin qubits
Source: Nat Commun. 2015 Nov 24;6:8955. doi: 10.1038/ncomms9955 (PMC4673878; doi:10.1038/ncomms9955)
Supplement: Supplementary Information — Supplementary Figures 1-6, Supplementary Table 1, Supplementary Notes 1-6 and Supplementary References [file ncomms9955-s1.pdf]

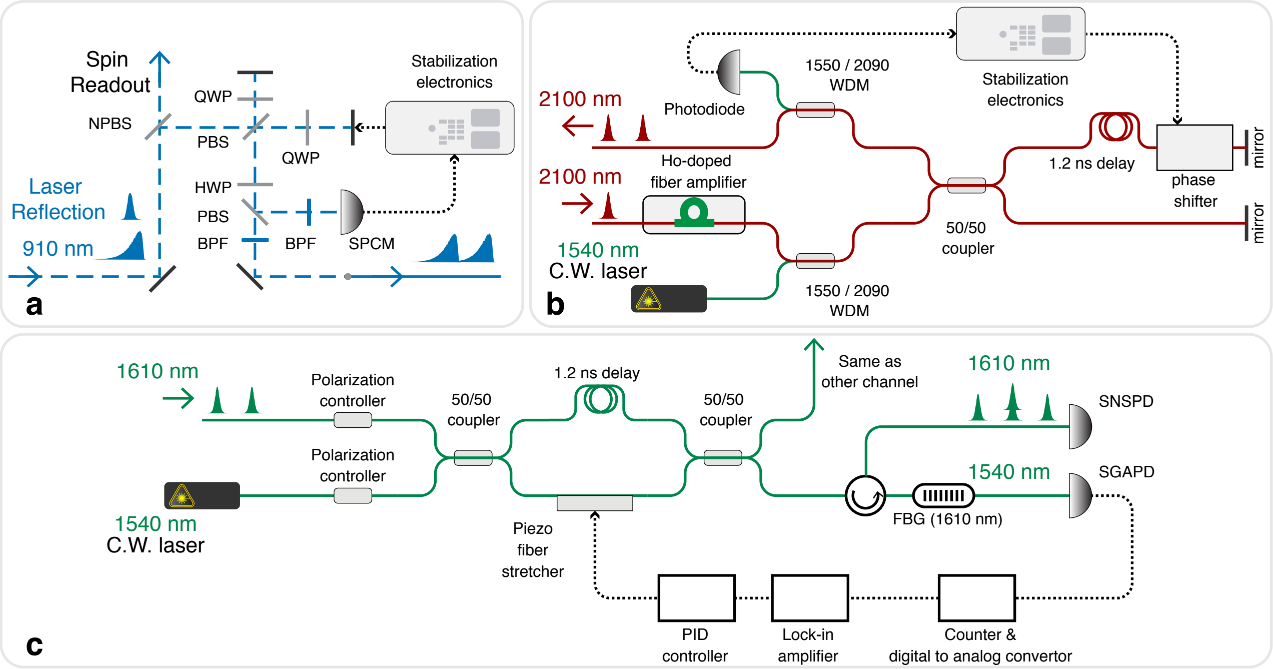

Supplementary Figure 1. **Actively stabilized interferometers for time-bin encoding. a, b, c,** Schematic diagrams of the unbalanced Michelson interferometers for **a,** Quantum-dot (QD) single photons; **b,** 2-μm generation; and **c,** downconverted 1.61-μm photons. **a,** (N)PBS: (non-)polarizing beamsplitter; HWP: half-wave plate; QWP: quarter-wave plate; BPF: bandpass filter; SPCM: single-photon counting module. **b,** C.W.: continuous-wave; WDM: wavelength-division multiplexer. **c,** FBG: fiber Bragg grating; SNSPD: superconducting nanowire single-photon detector; SGAPD: sinusoidally gated avalanche photodiode.

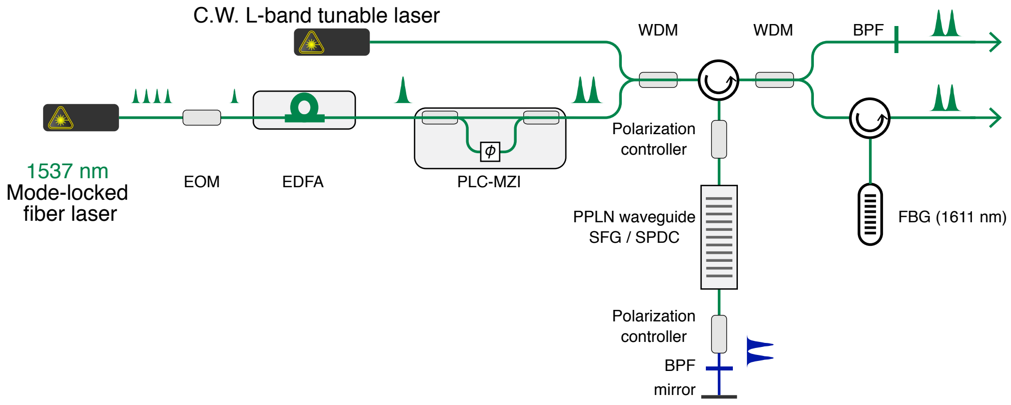

Supplementary Figure 2. **Schematic of the source of time-bin-entangled photon pairs based on periodically poled lithium niobate (PPLN) waveguides.** EOM: electro-optic modulator; EDFA: Erbium-doped fiber amplifier; PLC-MZI: planar lightwave circuit Mach-Zehnder interferometer; SFG: sum-frequency generation; SPDC: spontaneous parametric down-conversion.

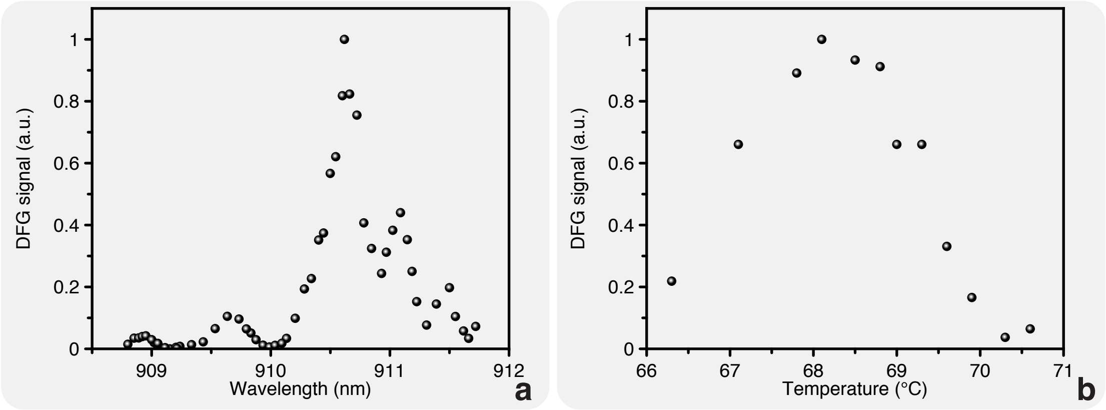

Supplementary Figure 3. **Optimization of difference-frequency generation efficiency. a,** Phase-matching curve of a PPLN waveguide taken by scanning the signal wavelength. **b,** Optimization of the difference-frequency generation (DFG) signal through temperature tuning.

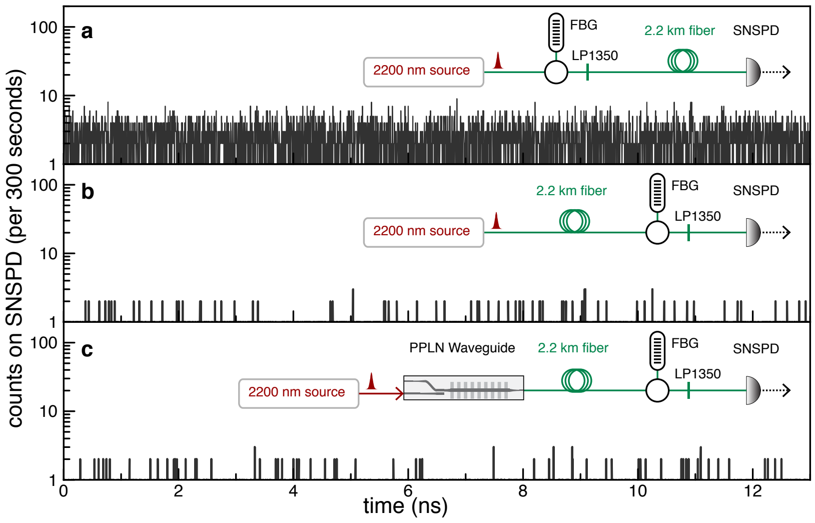

Supplementary Figure 4. **Residual background noise after downconversion.** In **a, b, c,** various filtering configurations are depicted in the insets. All measurements were performed with an average pump power of 3.1 mW exiting the pump source and a nominal pump-pulse duration of 30 ps. In **b** and **c**, the detected count rate was consistent with the dark count level of the detector. FBG: fiber Bragg grating.

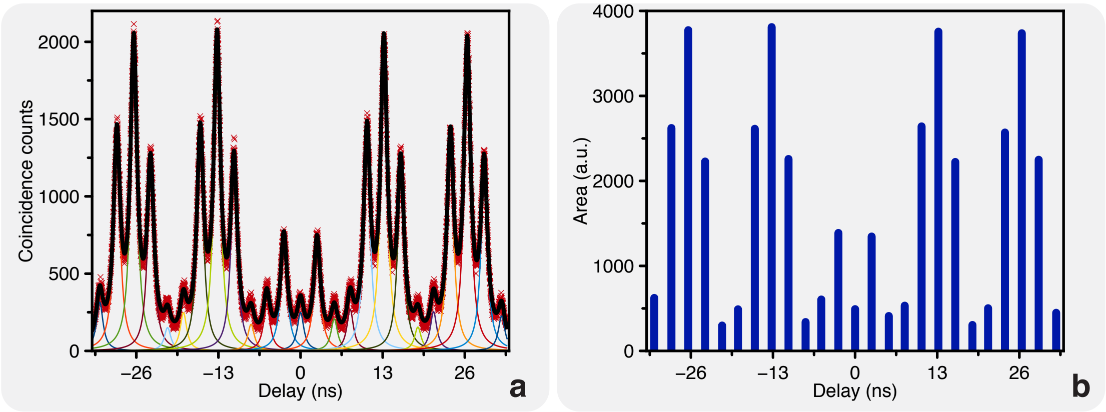

Supplementary Figure 5. **Extraction of the individual peak areas for two-photon interference between consecutive photons. a,** Fit to the histogram shown in the top panel of Fig. 5f as a series of peak functions. The red crosses represent the raw data points, whereas the bold black line represents the sum of the individual peak functions. **b,** Areas of the individual peak functions.

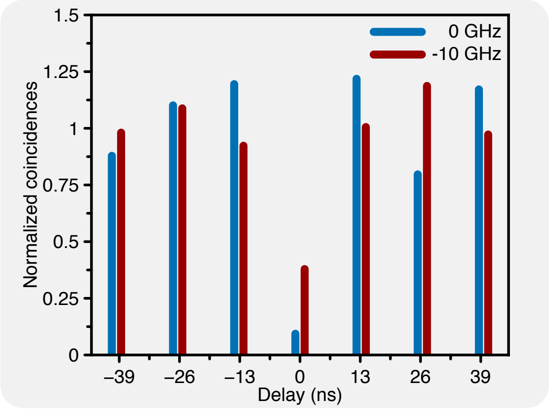

Supplementary Figure 6. **Representative histograms used to extract $n_{c,d}$ for two-photon interference between heterogeneous nodes. a,** at the frequency detuning of 0 GHz; **b,** at −10 GHz.

Light	Wavelength (nm)
Quantum dot	910.847
Diode laser	900.002
Ti:sapphire mode-locked laser	895.019
Telecom laser a	1561.210
Telecom laser b	1594.135
Pump pulse a	2097.465
Pump pulse b	2040.836
Target	1610.012

Supplementary Table 1. **Summary of the wavelengths used in the QD-laser two-photon interference experiment.** The pump-pulse and target wavelengths were not directly measured; instead, they were calculated based on energy conservation in the DFG processes.

Supplementary Note 1. Quantum states of light

In the low photon-number limit, a quantum state of light, with its photon wavepacket as $x(t)$, can be approximated as

$$|\psi\rangle = \left[\hat{1} + e^{i\phi_1}\sqrt{\bar{n}}\hat{a}_x^\dagger + e^{i\phi_2}\sqrt{g}(\bar{n}/2)\hat{a}_x^{\dagger 2}\right]|0\rangle\,, \tag{1}$$

where $\bar{n}$ is its mean photon number and g measures its two-photon probability relative to that of a Poissonian source. One can verify that $\langle\psi|\,\hat{a}_x^\dagger\hat{a}_x\,|\psi\rangle \sim \bar{n}$ so the choice of symbol $\bar{n}$ agrees with its physical meaning. Regarding g, particularly there are three illustrative cases:

$g = 0$: The state $|\psi\rangle$ essentially reduces to a single-photon state since the vacuum state is not detectable in photon counting.

$g = 1$: The state $|\psi\rangle$ becomes $|0\rangle + e^{i\phi_1}\sqrt{\bar{n}}\,|1\rangle + e^{i\phi_2}(\bar{n}/\sqrt{2})\,|2\rangle$, which is essentially a coherent state of $\alpha = \sqrt{\bar{n}}e^{i\phi_1}$ if $\phi_2 = 2\phi_1$. If not, the state still obeys the Poissonian distribution.

$g = 2$: The photon-number distribution of $|\psi\rangle$ becomes that of thermal light, since $P(1) = \bar{n}$ and $P(2) = \bar{n}^2$, where $P(i)$ is the probability of the i-th Fock state.

The photon wavepacket $x(t)$ contains a carrier wave and a slowly varying envelope. For example, the photon wavepacket of a Lorentzian type emitter has the form $x(t) = \mathrm{H}(t)e^{-i\omega t}e^{-\frac{\Gamma}{2}t}$, where $\mathrm{H}(t)$ is the Heaviside step function and Γ is the decay rate. Other common types of photon wavepacket envelopes include a Gaussian and a sech^2 type. Between two photon wavepackets $x(t)$ and $y(t)$, the mean wavepacket overlap, $V \equiv |\int dt x(t)y^*(t)|^2$, can be defined to measure the similarity of photons. V vanishes between photons unless they are identical in wavelength and wavepacket. Equivalently, one can also write $V = |\int d\omega X(\omega)Y^*(\omega)|^2$ because of Parseval's theorem, where $X(\omega)$ and $Y(\omega)$ are the Fourier transforms of $x(t)$ and $y(t)$, respectively. The photon wavepackets are equally well described in the frequency domain.

When the quantum states of light are not pure, the mean wavepacket overlap, which determines the two-photon interference visibility, can readily be shown to become $V = \mathrm{Tr}\,(\hat{\rho}_a\hat{\rho}_b)$, where $\hat{\rho}_x$ is the density operator of the quantum state x. The overlap V approaches 1 only when both $\hat{\rho}_a$ and $\hat{\rho}_b$ are pure states, in which case this generalization agrees with the original definition of V. We also mention that the mean wavepacket overlap differs from the commonly defined fidelity $F(\hat{\rho}_a, \hat{\rho}_b) \equiv \left(\mathrm{Tr}\left(\sqrt{\hat{\rho}_a^{1/2}\hat{\rho}_b\hat{\rho}_a^{1/2}}\right)\right)^2$ [1]. The overlap V concerns not only the similarity between but also the purity of the quantum states. For example, if $\hat{\rho}_a$ and $\hat{\rho}_b$ are the completely mixed states in an infinite-dimensional Hilbert space, then $F(\hat{\rho}_a, \hat{\rho}_b) = 1$ but $V = 0$. In general, $V \leq F$ holds.

Supplementary Note 2. Two-photon interference

In this section, we further discuss the possible causes of the finite mean wavepacket overlap that was observed in the two two-photon interference experiments, particularly with the spectral information provided in Fig. 6a. We attempt to develop a qualitative physical understanding of the observed non-idealities, acknowledging that a full quantitative analysis is not possible given the performed measurements. However, we also emphasize that our reported overlap V and visibility were directly extracted from the photon-counting measurements and do not rely on any specific association of dephasing mechanisms.

We first recall that the spectra of the downconverted quantum dot (QD) and laser photons were the wave-mixed outcomes of many light sources, which by themselves could have been frequency broadened for various reasons. To infer the overall spectrum after wave mixing, one could naively convolve all of the spectra of the individual light sources. Such a convolution was not accessible to us because no phase information was obtainable from the spectral-intensity measurements that we performed, but the underlying concept nevertheless hinted that the bandwidth of the overall spectrum would be predominantly determined by one light source if its bandwidth were much broader than those of the others. Concretely, the bandwidth of the downconverted laser photons was predominantly determined by the Ti:sapphire mode-locked laser because its bandwidth, 2.62 GHz if transform limited, was much broader than those of the telecom laser and the c.w. diode laser, whose bandwidths were on the order of 15 MHz and 1 MHz, respectively. Similarly, in the determination of the bandwidth of the downconverted QD photons, the lifetime-limited bandwidth of the QD photons, 0.234 GHz, was inconsequential because it was much narrower than 2.62 GHz. We therefore estimate that the Ti:sapphire mode-locked laser predominantly determined the downconverted laser spectrum and provided a

baseline for the downconverted QD spectrum, which could be further broadened by other dephasing mechanisms of the QD.

Regarding the downconverted laser spectrum, we note that its Gaussian-fitted bandwidth, 4.46 GHz, was broader than the transform-limited bandwidth, 2.62 GHz. It is known that the mode-locked laser model that we used (Spectra-Physics Tsunami), when configured with the 100-ps setting, produces a minimal bandwidth of 4 GHz, which yields a time-bandwidth product that is 1.27 times the transform limit. In our experiment, we deliberately operated the mode-locked laser at the GTI setting with the longest pulse duration as opposed to the setting with the minimal time-bandwidth product. Thus, the further broadening of the bandwidth to 4.46 GHz can conceivably be attributed to our GTI setting.

Regarding the downconverted QD spectrum, the dephasing mechanisms of the QD need to be considered in addition to those of the mode-locked laser. Practical quantum-dot single-photon sources do not generally exhibit perfect photon indistinguishability as a result of time jitter, pure dephasing, and spectral diffusion [2]. The time jitter process arises as a result of the finite relaxation rate of the intermediate state during non-resonant optical excitation, thus leading to an uncertainty in the start times of photon wavepackets [2]. This jitter, which is associated with phonon emission, occurs on a time scale of 10 ps [2]; therefore, its effect on photon indistinguishability can be neglected in our system because it is much shorter than the QD radiative decay time, 681 ps. The second cause, pure dephasing [2], may originate from the homogeneous broadening of the excited state [3]. This dephasing process can be most easily modeled as a random phase fluctuation $e^{i\phi(t)}$ that is added to the photon wavepacket. If we further assume that the dephasing process is stationary, independent, and identical over time, then we can write $\left\langle e^{i\phi(t)}e^{-i\phi(t+\tau)}\right\rangle = e^{-\alpha|\tau|}$, where α is a pure dephasing rate. Based on this model, we can calculate the mean wavepacket overlap to be [2]

$$V = \frac{\Gamma/2}{\alpha + \Gamma/2}, \tag{2}$$

where Γ is the QD radiative decay rate (0.234 GHz, inferred from an exponential decay time of 681 ps). In our test of the indistinguishability of consecutive photons from a QD, V was found to be 0.73 prior to downconversion. From Supplementary Equation (2), we can infer the pure dephasing rate α to be on the order of 0.043 GHz.

The third factor affecting photon indistinguishability, spectral diffusion, can be regarded as a process in which the QD emission line drifts slowly in wavelength because of charge fluctuations in the surrounding environment [4]. The spectral diffusion process also results in dephasing but occurs on a much longer time scale. If this time scale is much longer than the period of the optical excitation of the QD, then the spectral diffusion will have little effect on the overlap between the wavepackets of consecutive photons [4]. Indeed, by comparing the Gaussian-fitted bandwidths of the downconverted QD and laser photons (7.10 and 4.46 GHz, respectively), one can naively estimate that the QD bandwidth was Gaussian broadened from the lifetime limit to 5.52 GHz. Had this bandwidth broadening been caused not by spectral diffusion but by pure dephasing, the mean wavepacket overlap would have been limited to 0.04, and no photon indistinguishability among consecutive photons from the QD would have been observed in our test.

Supplementary Note 3. Toward spin-time-bin entanglement

Active phase stabilization

We present our solutions for the demonstration of spin-time-bin entanglement in this subsection. The primary task is to actively stabilize the phase relationship between the s- and l-bin photons as they traverse different arms of the interferometer during encoding, downconversion and readout. We explain in detail the configurations implemented for active phase stabilization of the interferometers.

We begin with the interferometer (Supplementary Fig. 1a) that was used to encode the QD photons. A single-frequency external-cavity diode laser (ECDL; Toptica DL pro 940) was used as a phase reference. To ensure that the ECDL light traversed the same optical path as did the QD photons, it was combined with the rotation laser in a fiber combiner before being launched into free space, and thus became σ^+-polarized. The ECDL wavelength was chosen to be 930 nm, which lay within the bandwidth of the downstream polarization optics, was sufficiently red detuned such that it would not excite the QD, could be easily filtered out because of the large wavelength separation from the QD, and could be separated using off-the-shelf bandpass filters. After passing through the unbalanced Michelson interferometer, half of the ECDL light exited the $-45°$ port and was isolated from the QD photons by a 930-nm BPF (Thorlabs FB930-10) before being detected by an SPCM (PerkinElmer SPCM-AQRH-14). The photocurrent intensity of the SPCM then indicated whether the OPL difference resulted in constructive interference. An all-in-one box containing stabilization electronics and a high-voltage amplifier (TEM Messtechnik GmbH Digital LaseLock with HV Amp) drove the piezo actuator (Thorlabs AE0505D16F) to compensate for any phase deviation. The SPCM was

chosen instead of a common photodiode to reduce the amount of ECDL light that was required for phase referencing to the nanowatt level; otherwise, the ECDL light would have been too strong to be removed by the BPF when entering the path for spin readout. Meanwhile, the ECDL light that exited the $+45^\circ$ port was also coupled into the PPLN waveguide but was not downconverted because its wavelength was far from phase matching. Using this setup, we demonstrated an interference visibility of greater than 99%.

For downconversion, the (2.1-μm) pump pulses were prepared in both time bins using another interferometer (Supplementary Fig. 1b), which was required to be stable in phase because this phase relationship would be imprinted on the downconverted photons. Because of the lack of availability of single-frequency 2.1-μm lasers, we used a telecom ECDL (New Focus Vidia 6427) as the phase reference and implemented the interferometer accordingly to accommodate both wavelengths. A Michelson configuration was chosen because the fiber splitter (Lightel PMC-S-22-5050-2090-0-L-2) were inherently narrowband and unevenly split ($\sim$ 30:70) at the phase-referencing wavelength. Consequently, the phase-reference light traversing the two arms of the interferometer combined at the photo-diode with nearly equal intensity ($R \cdot T = T \cdot R$) and interfered with good visibility. A fiber wavelength-division multiplexer (WDM; Lightel PMWDMS-12-1550-2090-0-L-2) combined the 2.1-μm light from the Ho-doped fiber amplifier and the telecom light from the ECDL. The fiber mirrors (WT&T FM-2D), which were gold coated at the fiber ends, reflected both the telecom and 2.1-μm light. The fiber phase shifter (General Photonics FPS-001-01-NT-PP-FC/APC), which essentially consisted of a section of fiber epoxied onto a piezo actuator, was also broadband; its short length (less than 2 cm) ensured minimal attenuation of the 2.1-μm light. The remaining phase-stabilization electronics were similar to those of the first interferometer. Using this setup, we confirmed an interference visibility of greater than 99%.

Another challenge facing the attempt to ensure a stable phase relationship between the s- and l-bin photons was that the (2.1-μm) pump pulses further suffered from wavelength instability originating from the seed Ti:sapphire mode-locked laser (Spectra-Physics Tsunami), which can drift by more than 50 pm in a hour and does not directly support wavelength stabilization. Wave-mixing this laser with a telecom ECDL (New Focus Vidia 6427) to generate the pump pulses, we were able to compensate for the wavelength drift of the former by modulating the drive current and grating angle of the latter. The resulting wavelength precision was determined by a laser wavemeter (Burleigh WA-1100).

Readout in the off-diagonal time-bin basis $\{|\mathrm{s}\rangle \pm |\mathrm{l}\rangle\}$ requires a third interferometer (Supplementary Fig. 1c) at 1.61 μm, where the low flux of the downconverted photons ($\sim$ 30,000 cps), in combination with the finite suppression ($\sim$ 40 dB) of the fiber Bragg grating (O/E Land) that served as a narrow-band filter, restricted the intensity of the phase-reference signal to a single-photon level. We detected this signal using sinusoidally gated avalanche photodiodes (SGAPD) [5], which proportionately generated avalanche pulses that were integrated using a LabView program. The integrated voltage was demultiplexed in a lock-in amplifier to determine the phase deviation, which was then compensated for by a PID controller (Toptica PID 110) driving a piezo actuator. Using this setup, we confirmed an interference visibility of greater than 99%.

Alternatives to polarization-to-time-bin encoding

We recall that frequency conversion based on a PPLN waveguide is generally polarization-dependent and investigate alternative solutions to this problem. The polarization dependence of PPLN waveguides results both from the highly anisotropic nonlinear susceptibility of $LiNbO_3$ and, for proton-exchange devices, that the waveguides support only a single polarization [6]. One obvious solution, i.e., downconverting H and V photons in two separate PPLN waveguides [7, 8], yields two separate optical paths, each consisting of heterogeneous medium including free space, optical fibers and PPLN waveguides. Active stabilization of the difference between the two paths therefore brings more complexity, to offset which could incur additional engineering of PPLN waveguides [6]. Another solution utilizes a polarization Sagnac interferometer to ensure that photons of orthogonal polarization traverse the same optical path [9, 10]. But the three well-separated wavelengths ($\{\lambda_\mathrm{s}, \lambda_\mathrm{t}, \lambda_\mathrm{p}\}$) in the downconversion process could hardly be supported altogether by commercially available fiber-optic PBSs and circulators that are used in the polarization Sagnac interferometer. Since these alternative solutions require nontrivial engineering of frequency-conversion and/or fiber-optic devices, we implemented polarization-to-time-bin encoding to circumvent the polarization dependence of PPLN waveguides.

Supplementary Note 4. Background noise

The key step in our demonstration of background-free downconversion was to determine the origins of the spurious nonlinear processes, which are summarized in this section. A major non-ideality in the frequency-conversion setup was the QPM random duty-cycle (RDC) error. The employed electric field poling procedure in this work yielded a random deviation from the designed duty cycle of approximately 10% [11]. For three-wave mixing processes,

these errors resulted in a conversion-efficiency pedestal that was independent of the phase-matching conditions. One consequence of the QPM RDC errors was the process of spontaneous parametric fluorescence (SPF) [12]. Parametric fluorescence is the spontaneous splitting of a pump photon into a lower-energy signal/idler pair with energies of $\omega_{\mathrm{s}} + \omega_{\mathrm{i}} = \omega_{\mathrm{p}}$ when phase matched. However, the QPM efficiency pedestal gives rise to the generation of a "white-noise"-like collection of photons at all frequencies $\omega < \omega_{\mathrm{p}}$. We can, however, prevent SPF from overlapping with the signal photons in frequency by judiciously employing long-wave-pumped downconversion [13]. As a counterexample, in short-wave-pumped downconversion, the pump wavelength would be selected to lie at approximately 580 nm; in this case, the SPF would span both the signal and target wavelengths and become nearly impossible to reject.

Another consequence of the QPM RDC errors was parasitic pump second-harmonic radiation created at $\lambda_{\mathrm{p}}/2$ [14]. As a practical example, when reaching peak downconversion with our DFG PPLN waveguide, we observed the power level of the parasitic pump SHG to be approximately −60 dBm, roughly consistent with a 10^{-5} efficiency suppression from a truly phase-matched SHG process. Because the wavelength of this SHG component was known (approximately 1.05 μm) and was well separated from the target wavelength, it could be straightforwardly absorbed by a filter on a Si substrate, whose bandgap is approximately 1.1 μm.

A third origin of noise was spontaneous Raman scattering [13]. In general, Raman scattering involves the scattering of a photon off an optical phonon in a material, causing the photon to either redshift through the creation of a phonon (so-called Stokes scattering) or blueshift through the absorption of a phonon (anti-Stokes scattering). In our case, one would naively anticipate that the Raman photons should originate mostly in, and can be rejected by avoiding, the fingerprint Raman peaks of $LiNbO_3$, which are located at frequency shifts of approximately 252 and 625 cm^{-1}. For a target wavelength of 1.55 μm, these peaks correspond to pump wavelengths of 1.61 and 1.71 μm in long-wave-pumped downconversion. However, previous studies have suggested the presence of a Raman pedestal that exhibits a slower roll-off with frequency than would be expected from the sum of Lorentzian peaks [13]. Long-wave-pumped downconversion was again beneficial in rejecting this pedestal because it enabled the containment of the Raman photons on the side corresponding to anti-Stokes scattering, which exhibited an additional roll-off in frequency following a Boltzmann distribution. Then, because of the broadband nature of the Raman photons, they could be further rejected to a very low level using fiber Bragg gratings with bandwidths of 1–2 nm.

With the above filtering strategies applied, the noise-count probability per pump pulse was reduced to the level of 10^{-8} in our previous study [14], in which we assumed that the remaining background noise was caused by Raman scattering, as discussed above, that was not completely rejected by the fiber Bragg gratings. However, we later discovered that the noise persisted at the same level even when the PPLN waveguide was not present, as shown in Supplementary Fig. 4a. We thus reasoned that this remaining background noise was generated by the high-peak-power pump pulses in the downstream optics of the waveguide, which consisted of filtering components such as a fiber Bragg grating and a Si-substrate filter. We suspect that the noise photons originated from the Raman scattering in the SMF-28 fibers or the fluorescence in the Si substrate. Regardless, the noise could be removed by attenuating the pump pulses after the waveguide but before the filtering components using a long, coiled SMF-28 fiber (not SMF-28e), as shown in Supplementary Fig. 4b. Consequently, we could demonstrate a noise-count probability per pump pulse of less than 10^{-11}, even when the PPLN waveguide was present, as shown in Supplementary Fig. 4c.

Cascaded downconversion

Cascaded downconversion helps to ensure that the necessary condition for background-free downconversion, namely, that the pump wavelength is sufficiently longer than the target wavelength, can be fulfilled. As an example, downconversion of a signal wavelength at 780 nm can lead to nearly degenerate pump and target wavelengths at approximately 1560 nm. Indeed, all previous attempts [15–17] have resulted in finite background noise. We propose that a two-step downconversion process pumped at 3.12 μm can resolve this issue. In this two-step process, the target wavelengths will subsequently be 1.04 and 1.56 μm, well separated from the pump wavelength. The required pump wavelength, 3.12 μm, can be generated in a DFG setup that is similar to that depicted in Fig. 4a, except that the telecom C/L-band laser should be replaced with a telecom O-band laser.

Supplementary Note 5. Toward long-distance entanglement swapping

Our system may further be combined with a source of time-bin-entangled photon pairs to demonstrate long-distance entanglement swapping. In the midpoint-source scheme [18], an entangled photon pair is generated at the midpoint between two quantum dots, and takes the following form in the time-bin basis:

$$|\varphi\rangle = \frac{1}{\sqrt{2}} \left(|\mathrm{s_s}; \mathrm{s_i}\rangle + |\mathrm{l_s}; \mathrm{l_i}\rangle \right), \quad (3)$$

where the subscripts s and i denote the signal and idler photons, respectively, that are individually sent to two QDs. In addition, recall that the entangled spin-photon pair from a QD is encoded into the time-bin format: $|\psi\rangle = \frac{1}{\sqrt{2}}\left(|\uparrow\rangle \otimes |i\mathrm{l}\rangle + |\downarrow\rangle \otimes |\mathrm{s}\rangle\right)$. The joint state of the spin-photon and photon-photon entangled pair then becomes

$$\begin{aligned} |\psi\rangle \otimes |\varphi\rangle = \frac{1}{2\sqrt{2}}(& |\Phi^{+}\rangle \left(|\uparrow;\mathrm{s_s}\rangle + |\downarrow;\mathrm{l_s}\rangle\right) + |\Phi^{-}\rangle \left(|\uparrow;\mathrm{s_s}\rangle - |\downarrow;\mathrm{l_s}\rangle\right) \\ + & |\Psi^{+}\rangle \left(|\uparrow;\mathrm{l_s}\rangle + |\downarrow;\mathrm{s_s}\rangle\right) + |\Psi^{-}\rangle \left(|\uparrow;\mathrm{l_s}\rangle - |\downarrow;\mathrm{s_s}\rangle\right)), \end{aligned} \quad (4)$$

where the Bell states $|\Psi^{\pm}\rangle = \left(|\mathrm{sl}\rangle \pm |\mathrm{ls}\rangle\right)/\sqrt{2}$ and $|\Phi^{\pm}\rangle = \left(|\mathrm{ss}\rangle \pm |\mathrm{ll}\rangle\right)/\sqrt{2}$ physically consist of the QD photon and the idler photon. A Bell state measurement (BSM) in the $|\Psi^{-}\rangle$ state projects the joint state as $\left(|\uparrow;\mathrm{l_s}\rangle - |\downarrow;\mathrm{s_s}\rangle\right)/\sqrt{2}$: entanglement is established between the QD spin and the signal photon from the entangled photon-pair source. Note that the BSM takes place adjacent to the QD (Fig. 1); whenever the BSM fails due to photon loss, the QD can be re-initialized to generate the next entangled spin-photon pair as soon as possible. The signal photon, however, propagates over long distances toward the other end of the quantum channel. Eventually, another BSM establishes the entanglement between two QD spins.

The protocol to demonstrate entanglement swapping begins with a successful BSM, which results from two-photon interference at a beam splitter. Among the successful coincidence patterns in the time-bin basis (in Fig. 1), only that of $|\Psi^{-}\rangle$ can be identified in practice because of the finite dead time (on the order of 30 ns) of single-photon detectors. A successful detection then not only projects the joint state of the entangled spin-photon and photon-photon pairs but also heralds the arrival of the signal photon from the entangled photon-pair source. To verify that the entanglement has been swapped, this signal photon is measured concurrently in the off-diagonal basis $\{|\mathrm{s}\rangle + |\mathrm{l}\rangle, |\mathrm{s}\rangle - |\mathrm{l}\rangle\}$ using both output ports of a stabilized interferometer (Supplementary Note 3). The correlations of these measurements with the spin readout in the $\{|\uparrow\rangle \pm |\downarrow\rangle\}$ basis yield the conditional probability and, subsequently, the entanglement fidelity [8]. Although the verification requires four-fold coincidences, the heralding nature of entangled photon-pair sources effectively reduces this requirement to three-fold coincidences times the heralding efficiency.

A source of time-bin-entangled photon pairs based on PPLN waveguides

We designed a source of time-bin-entangled photon pairs that is efficient, robust and alignment free. This design is based on telecom lasers, fiber components, and a single pigtailed PPLN waveguide that provides both sum-frequency generation (SFG) and spontaneous parametric downconversion (SPDC). Essentially, time-bin-entangled photon pairs are generated by pumping the PPLN waveguide with coherently superposed consecutive laser pulses, between which the time delay is longer than the coherence time of the generated photons [19, 20].

Our design is schematically illustrated in Supplementary Fig. 2. A fiber mode-locked laser (Calmar optics) generates a 10-ps pulse train at a repetition rate of 9.55 GHz, which is pulse picked to 75.76 MHz using a fiber EOM (Lucent 2623NA). The power of this train is boosted in an EDFA. Each pulse is split and recombined in a planar lightwave circuit Mach-Zehnder interferometer (PLC-MZI; NTT) to form coherently-superposed consecutive pulses. The resulting pulse train at 1537 nm is combined with the c.w. light from a telecom laser at 1605 nm in a telecom C/L-band WDM and wave-mixed in the PPLN waveguide, which supports only one photon polarization. Consecutive pulses at 785 nm are then generated through sum-frequency generation and are subsequently reflected back into the PPLN waveguide to pump the SPDC process. The original telecom laser light is removed by double-passing it through a 785-nm bandpass filter (Omega; $>$ 50 dB rejection at 1.5 μm), thus producing clean time-bin-entangled photon pairs at the output of the PPLN waveguide.

The signal and idler photons of the entangled photon pairs are separated in two steps: first, a telecom C/L-band WDM separates the C-band and L-band photons; and then, an FBG serving as a narrow-band filter on the L-band branch (heralding branch) isolates the photons that match the downconverted QD photons in terms of wavelength. These isolated photons constitute the idler photons. For two-photon interference, the bandwidth of the FBG is chosen in accordance with that of the downconverted QD photons, whose bandwidth is determined by the bandwidth of the 30-ps or 100-ps pulses generated by the Ti:sapphire mode-locked laser. Although the center wavelength of the FBG has a limited tuning range, the tunability of the (2.1-μm) pump pulses eliminates any wavelength mismatch. Optimal wavepacket overlap between the idler photon and the downconverted QD photon is therefore ensured. We expect that upon the detection of the idler photon, the signal photon at the C-band branch can be heralded at a 32% efficiency using this setup.

Signal and noise analysis

As a case study, we analyze the signal-to-noise ratio involved in establishing an entangled spin-photon pair at long distances. We define the following symbols: p_{spin} is the probability of successfully detecting a photon for spin readout, p_{photon} is the probability of detecting a downconverted QD photon, p_0 is the probability of detecting the idler photon from the entangled-photon-pair source, and η is the heralding efficiency of the signal photon upon detection of the idler photon. Using the above definitions, the probability of a successful entanglement-swapping event is given by $\frac{1}{8}p_{\mathrm{spin}}p_{\mathrm{photon}}p_0\eta$.

The dominant noise source in this case is the two-pair generation events of the entangled photon-pair source. As a classical process, SPDC gives rise to two-pair generation at a probability of p_0^2. Consequently, the consecutive pump pulses for SPDC generate the following two-pair state:

$$\frac{1}{\sqrt{2}}\left(|2;0\rangle_{\mathrm{s}}|2;0\rangle_{\mathrm{i}} + 2|1;1\rangle_{\mathrm{s}}|1;1\rangle_{\mathrm{i}} + |0;2\rangle_{\mathrm{s}}|0;2\rangle_{\mathrm{i}}\right). \tag{5}$$

Here, $|n;m\rangle$ denotes the n (m) photon state in the s (l) time bin and the subscripts s and i denote signal and idler. The two-pair state $|1;1\rangle_{\mathrm{i}}$ has a 1/2 probability of producing the same detection pattern as that of the state $|\Psi^-\rangle$, causing a false coincidence in the BSM. The upper bound on the probability of such false events is $\frac{1}{4}p_{\mathrm{spin}}p_0^2\eta$.

Thus, the lower bound on the signal-to-noise ratio due to two-pair generation events is

$$\frac{\mathrm{S}}{\mathrm{N}} \geq \frac{p_{\mathrm{photon}}}{2p_0}. \tag{6}$$

Naively, the overall coincidence count rate can be increased by operating at a higher pump laser power to increase p_0. However, a good signal-to-noise ratio can be maintained only if $p_0 \ll p_{\mathrm{photon}}$. Multi-pair generation sets the limit on the photon flux of the SPDC source to below the downconverted QD photon flux. This limit may be circumvented given recent progress in generating entangled photon pairs using QDs [21, 22]. A trade-off can be made between system complexity and communication rate.

Supplementary Note 6. PPLN waveguides: design and characterization

We implemented guided-wave structures in PPLN based on reverse proton exchange (RPE) for their following features: high conversion efficiency, low propagation losses, and low insertion loss when pigtailed to standard optical fibers (e.g., SMF-28). The fiber pigtailing further provided long-term stability and rigidity that are essential for single-photon experiments. In addition, the mature fabrication process allowed us to implement various integrated waveguide structures that are shown in Fig. 4a. These integrated structures were essential for efficient wave-mixing and light-coupling of the waveguides. Concretely, at both input ports of the device were a mode filter and an adiabatic taper. Since the spacing between the input ports was chosen to match that of the fiber V-groove array for pigtailing, an S-bend structure was necessary to bring one input channel into proximity to the other. A directional coupler then followed to evanescently couple the pump light into the adjacent signal channel. The combined pump and signal light then entered the quasi-phase-matching (QPM) mixing region. At the output of the mixing region, the downconverted light at telecom wavelengths was sent through another mode conditioner before being coupled into an optical fiber (SMF-28) pigtailed to the exit facet of the waveguide.

We tested the phase-matching condition of the waveguide by measuring the device's small-signal transfer function as shown in Supplementary Fig. 3a, where the waveguide was held at 68 °C. To acquire such data, the DFG power was monitored when the wavelength of a 910-nm laser was scanned and that of the 2-μm pump light was fixed. For efficient downconversion of the QD photons, the peak wavelength of the phase-matching curve should coincide with the QD wavelength. This can be accomplished by fine tuning the device temperature to ensure that the maximal DFG is achieved, as illustrated in Supplementary Fig. 3b.

Additionally, we found the phase-matching curve in Supplementary Fig. 3a deviating from the $\mathrm{sinc}^2\left(\frac{\Delta k L}{2}\right)$ form of an ideal waveguide poled in a periodic pattern alternating in a square wave fashion, where Δk is the phase mismatch and L is the length of the wave-mixing region. The asymmetry of the curve suggested that some properties of the waveguide (refractive index, temperature, etc.) were not uniform along the propagation direction [23]. In our setup, the waveguide was likely heated uniformly along its length due to the rod-shaped heater sitting side-by-side underneath the waveguide. We suspect the asymmetry results from waveguide fabrication errors, including nonideality in lithography and an offset from the so-called noncritical design [24].

Supplementary References

[1] Nielsen, M. A. & Chuang, I. L. *Quantum Computation and Quantum Information* (Cambridge University Press, Cambridge, 2000).

[2] Santori, C., Fattal, D., Vuckovic, J., Solomon, G. S. & Yamamoto, Y. Single-photon generation with InAs quantum dots. *New Journal of Physics* **6**, 89–89 (2004).

[3] He, Y.-M. *et al.* On-demand semiconductor single-photon source with near-unity indistinguishability. *Nature nanotechnology* **8**, 213–7 (2013).

[4] Santori, C., Fattal, D., Vučković, J., Solomon, G. S. & Yamamoto, Y. Indistinguishable photons from a single-photon device. *Nature* **419**, 594–597 (2002).

[5] Namekata, N., Adachi, S. & Inoue, S. 1.5 GHz single-photon detection at telecommunication wavelengths using sinusoidally gated InGaAs/InP avalanche photodiode. *Optics express* **17**, 6275–82 (2009).

[6] Langrock, C., Kumar, S., McGeehan, J., Willner, A. & Fejer, M. All-optical signal processing using $\chi^{(2)}$ nonlinearities in guided-wave devices. *Journal of Lightwave Technology* **24**, 2579–2592 (2006).

[7] Takesue, H., Diamanti, E., Langrock, C., Fejer, M. M. & Yamamoto, Y. 1.5-μm single photon counting using polarization-independent up-conversion detector. *Optics Express* **14**, 13067–72 (2006).

[8] De Greve, K. *et al.* Quantum-dot spin-photon entanglement via frequency downconversion to telecom wavelength. *Nature* **491**, 421–5 (2012).

[9] Kim, T., Fiorentino, M. & Wong, F. N. C. Phase-stable source of polarization-entangled photons using a polarization Sagnac interferometer. *Physical Review A* **73**, 012316 (2006).

[10] Asobe, M., Miyazawa, H., Tadanaga, O., Nishida, Y. & Suzuki, H. A highly damage-resistant Zn:LiNbO3 ridge waveguide and its application to a polarization-independent wavelength converter. *IEEE Journal of Quantum Electronics* **39**, 1327–1333 (2003).

[11] Pelc, J. S., Phillips, C. R., Chang, D., Langrock, C. & Fejer, M. M. Efficiency pedestal in quasi-phase-matching devices with random duty-cycle errors. *Optics letters* **36**, 864–6 (2011).

[12] Pelc, J. S., Langrock, C., Zhang, Q. & Fejer, M. M. Influence of domain disorder on parametric noise in quasi-phase-matched quantum frequency converters. *Optics Letters* **35**, 2804–2806 (2010).

[13] Pelc, J. S. *et al.* Long-wavelength-pumped upconversion single-photon detector at 1550 nm: performance and noise analysis. *Opt. Express* **19**, 21445–21456 (2011).

[14] Pelc, J. S. *et al.* Downconversion quantum interface for a single quantum dot spin and 1550-nm single-photon channel. *Optics express* **20**, 27510–9 (2012).

[15] Ikuta, R. *et al.* Wide-band quantum interface for visible-to-telecommunication wavelength conversion. *Nature Communications* **2**, 1544 (2011).

[16] Ikuta, R. *et al.* High-fidelity conversion of photonic quantum information to telecommunication wavelength with superconducting single-photon detectors. *Physical Review A* **87**, 010301 (2013).

[17] Albrecht, B., Farrera, P., Fernandez-Gonzalvo, X., Cristiani, M. & de Riedmatten, H. A waveguide frequency converter connecting rubidium-based quantum memories to the telecom C-band. *Nature communications* **5**, 3376 (2014).

[18] Jones, C., De Greve, K. & Yamamoto, Y. A high-speed optical link to entangle quantum dots. Preprint at http://arxiv.org/abs/1310.4609v1 (2013).

[19] Tanzilli, S. *et al.* PPLN waveguide for quantum communication. *The European Physical Journal D - Atomic, Molecular and Optical Physics* **18**, 155–160 (2002).

[20] Zhang, Q. *et al.* Generation of 10-GHz clock sequential time-bin entanglement. *Optics express* **16**, 3293–8 (2008).

[21] Müller, M., Bounouar, S., Jöns, K. D., Glässl, M. & Michler, P. On-demand generation of indistinguishable polarization-entangled photon pairs. *Nature Photonics* **8**, 224–228 (2014).

[22] Jayakumar, H. *et al.* Time-bin entangled photons from a quantum dot. *Nature communications* **5**, 4251 (2014).

[23] Helmfrid, S., Arvidsson, G. & Webjörn, J. Influence of various imperfections on the conversion efficiency of second-harmonic generation in quasi-phase-matching lithium niobate waveguides. *Journal of the Optical Society of America B* **10**, 222–229 (1993).

[24] Chang, D. *et al.* Complex-transfer-function analysis of optical-frequency converters. *Optics Letters* **39**, 5106–5109 (2014).
